# Genome-Wide Identification of Long Noncoding RNA and Their Potential Interactors in *ISWI* Mutants

**DOI:** 10.3390/ijms23116247

**Published:** 2022-06-02

**Authors:** Ludan Zhang, Shuai Zhang, Ruixue Wang, Lin Sun

**Affiliations:** Beijing Key Laboratory of Gene Resource and Molecular Development, College of Life Sciences, Beijing Normal University, Beijing 100875, China; 202021200053@mail.bnu.edu.cn (L.Z.); 202031200005@mail.bnu.edu.cn (S.Z.); 202021200019@mail.bnu.edu.cn (R.W.)

**Keywords:** ISWI, chromatin remodel, long non-coding RNA (lncRNA), transposable elements, embryo TSA-FISH

## Abstract

Long non-coding RNAs (lncRNAs) have been identified as key regulators of gene expression and participate in many vital physiological processes. Chromatin remodeling, being an important epigenetic modification, has been identified in many biological activities as well. However, the regulatory mechanism of lncRNA in chromatin remodeling remains unclear. In order to characterize the genome-wide lncRNA expression and their potential interacting factors during this process in *Drosophila*, we investigated the expression pattern of lncRNAs and mRNAs based on the transcriptome analyses and found significant differences between lncRNAs and mRNAs. Then, we performed TSA-FISH experiments of candidate lncRNAs and their potential interactors that have different functions in *Drosophila* embryos to determine their expression pattern. In addition, we also analyzed the expression of transposable elements (TEs) and their interactors to explore their expression in *ISWI* mutants. Our results provide a new perspective for understanding the possible regulatory mechanism of lncRNAs and TEs as well as their targets in chromatin remodeling.

## 1. Introduction

ISWI is a member of the SWI2/SNF2 family of chromatin remodeling factors and is found in several different chromatin remodeling complexes in *Drosophila* [1]. In vivo, ISWI complexes are involved in the regulation of important physiological activities, such as gene transcription, heterochromatin formation, and X chromosome inactivation [2]. Among the six known ISWI complexes in *Drosophila*, NURF, NoRC, and ToRC are significantly involved in transcriptional activation, whereas RSF, ACF, and CHRAC use their nucleosome remodeling activity to close gaps in the nucleosome array during chromatin assembly or after division, thereby improving chromatin fiber stability and foldability [3]. So far, all ISWI complexes that have been studied change their nucleosome positions by repositioning histone octamers along DNA. This process is called nucleosome sliding [4]. Studies have demonstrated that one of ISWI’s complexes, NURF, can reshape nucleosomes to activate genes in chromatin, and it has been suggested that its functions may be particularly needed during transcriptional initiation [5]. The loss of ACF1, a subunit of the ISWI complex ACF, can seriously affect ovarian development and lead to tumor formation [3]. However, how ISWI complexes target and regulate biological processes remain largely unknown.

Long non-coding RNAs (lncRNAs) usually refer to non-coding RNA transcripts with lengths of more than 200 nt, high spatiotemporal specificity, and a conserved secondary structure [6]. New evidence suggests that despite their low coding potential, lncRNAs play roles in many cellular processes, including gene regulation, apoptosis, and embryonic development. Four functional types of lncRNA, including signal, bait, guidance and support, have been identified [7]. Based on the genomic location of these lncRNAs, they can be divided into four groups: intronic-lncRNAs generated in gene intron regions, lincRNAs generated between two genes, sense-lncRNAs generated in the sense chain, and antisense lncRNAs generated in the gene antisense chain [8]. In general, lncRNAs affect gene expression at three main levels: chromatin regulation, transcriptional regulation, and post-transcriptional regulation [9,10]. Many lncRNAs localize to chromatin to interact with proteins to promote or inhibit their binding and activity in target DNA regions [9,11]. The relative position of lncRNAs and their neighboring genes are key determinants of their regulatory relationships. Since lncRNA transcription has been found to be evolutionarily conserved, the genomic distribution of lncRNAs can represent evolutionary adaptations of genes [9,12]. In addition to their roles in transcriptional regulation and nuclear organization, lncRNAs control several other aspects of gene expression. However, lncRNAs function primarily through their ability to establish interactions with proteins and nucleic acids [9,13]. Compared with human and mouse lncRNAs, *Drosophila* lncRNAs have been studied less. Some important lncRNAs have been found to be associated with the *Drosophila* male-specific lethal (MSL) complex. LncRNA roX1 and roX2 are components of the MSL complex, that assist MSL2 in binding to other subunits of the complex and target the X chromosome in male *Drosophila* [14].

Studies have shown that there are some sequence similarities between lncRNAs and transposable elements (TEs) [15]. This similarity can be explained because they evolutionarily derive from TEs. TEs are repetitive, movable genomic sequences with the ability to integrate into new sites in the genome [16]. Transposon activation is critical at different developmental stages [17]. The simplest mechanism by which TEs can cause chromosomal rearrangements is through participation in an ectopic recombination event [18]. They are ubiquitous in all living organisms, including plants and animals, and make up 45% of the human genome and at least 80% of the maize genome [19,20,21]. The *Drosophila* melanogaster genome is one of the best studied eukaryotic genomes and while only about 20% of the genome consists of TEs, at least 30% of these elements are full length and believed to be active [22,23]. In addition to their sequence similarity, studies have confirmed that lncRNA embedded-TEs can regulate the expression, localization, and tissue specificity of lncRNA [24].

In this study, we explored the possible relationship between ISWI and lncRNAs. Based on the transcriptome sequencing results of *ISWI* mutant and wild-type *Drosophila*, we identified the global long non-coding RNAs and their potential interactors in chromatin remodeling complexes, and screened some TEs that might be involved as well. In addition, the expression patterns of some lncRNAs and their co-expressed genes were investigated through embryonic TSA-FISH experiments.

## 2. Results

### 2.1. Hybridization and Identification of ISWI Mutation Lines

**Figure 1:**
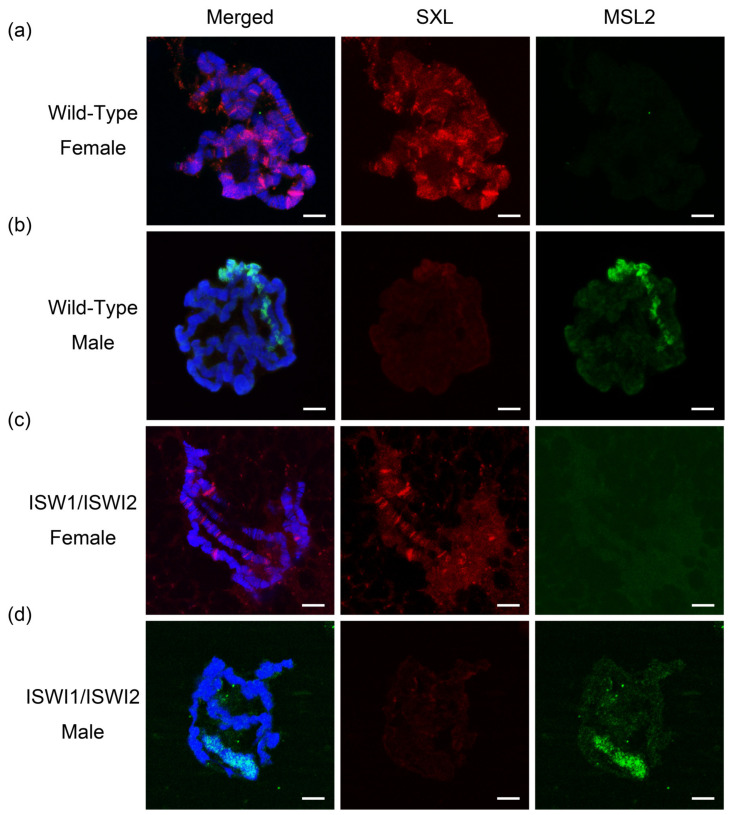
Immunofluorescence of *Drosophila* polytene chromosomes. Immunofluorescence of *Drosophila* polytene chromosomes from third instar larvae of wild-type female (**a**), wild-type male (**b**), ISWI1/ISWI2 female (**c**) and ISWI1/ISWI2 male (**d**). The red channel is the signal from SXL and the green channel is the signal from MSL2. DNA is stained with DAPI in blue. Scale bars, 5 μm.

Homozygous *ISWI* mutations lead to the death of *Drosophila* male adults, so we performed genetic hybridization with two ISWI point mutation strains (*y w*; *ISWI1/CyO·GFP*, *y w*; *ISWI2/CyO·GFP*). Both ISWI1 and ISWI2 are located on chromosome 2 with green fluorescent protein GFP on the balancer chromosome. We performed immunofluorescence staining of male and female *Drosophila* third instar larvae salivary gland chromosomes (Figure 1).

The *Drosophila* RNA-binding protein Sex-lethal (Sxl) controls female development [25]. In female *Drosophila*, SXL undergoes alternative splicing. Active SXL affects the synthesis of the downstream MSL2 protein. In male flies, the exon 3 of SXL (including the terminator) is not spliced, SXL is inactivated, and the downstream MSL2 protein can be synthesized normally. MSL2 recruits other components around the X chromosome to form the MSL complex targeting the X chromosome of male *Drosophila* [26]. Salivary gland polytene chromosome results showed a clear red SXL signal and no green MSL2 protein antibody signal in the females of wild-type and *ISWI* mutant samples (Figure 1a,c). In contrast, the green MSL2 protein signal was evident in male flies and enriched on the X chromosome (Figure 1b). Compared with wild-type male *Drosophila*, the X chromosome was shown to be significantly decondensed and shortened in *ISWI* mutant males (Figure 1d). This morphologic change of X chromosome with bouts phenotype in male Drosophila is found to be characteristic of ISWI mutants [27]. The results showed that the mutant strain ISWI1/ISWI2 was correctly constructed.

### 2.2. Identification and Genomic Characteristics of lncRNAs and mRNAs

**Figure 2:**
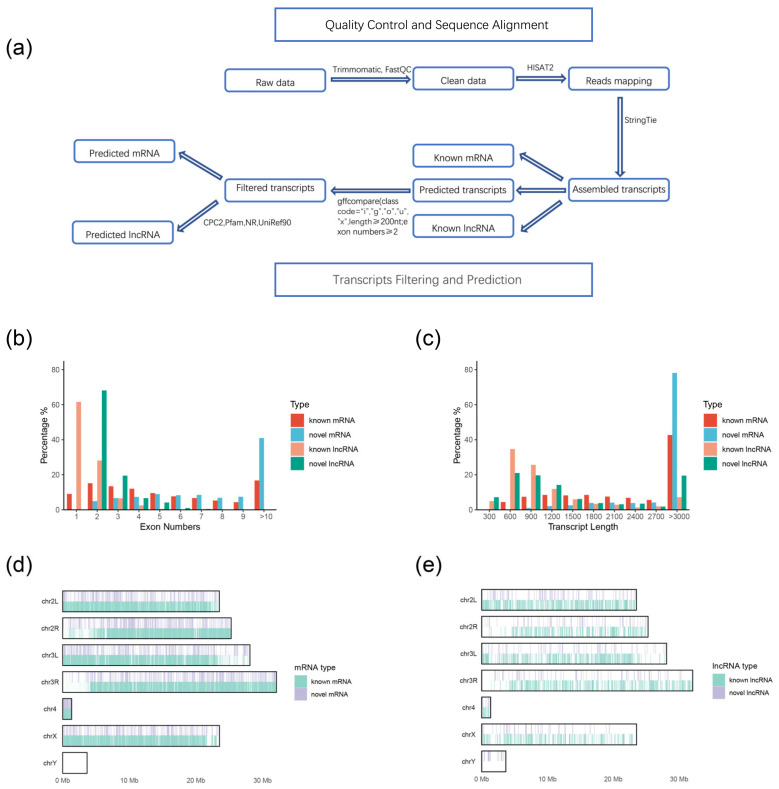
Identification and genomic characteristics of mRNAs and lncRNAs (**a**) Overview of the identification pipeline for putative mRNAs and lncRNAs in *ISWI* mutant *Drosophila*. (**b**) Distribution of exon numbers in mRNAs and lncRNAs. (**c**) Distribution of transcript length in mRNAs and lncRNAs. (**d**) The number distribution of mRNAs on the chromosome. (**e**) The number distribution of lncRNAs on the chromosome.

The third instar larvae of wild-type and *ISWI* mutants were collected, and three RNA replicates from every group were extracted and sequenced. We used raw data to obtain lncRNAs and mRNAs through assembly and prediction methods as shown in Figure 2a (explained in detail in the Section 4).

First, we compared transcripts with known genes and lncRNAs in the *Drosophila* genome database, and found 27,656 known mRNA transcripts and 1859 known lncRNA transcripts. The predicted transcripts were filtered by exon number and transcript length (exon number ≥ 2, length ≥ 200 nt). Next, we analyzed the coding capacity of the filtered transcripts using the CPC tool and retained 600 possible lncRNA transcripts. We also scanned each transcript according to a six-class protein framework, and 5369 mRNA transcripts with coding function were found. We refer to these assembled transcripts as novel lncRNAs and mRNAs.

To characterize the mRNA and lncRNA transcripts, we analyzed the number of exons and the transcription length of each transcript (Figure 2b,c). It was found that the number of novel lncRNA exons was higher than the number of known lncRNA exons. Most of the known lncRNAs were found to have only one exon, while the number of novel lncRNA exons was mostly 2, and there were novel lncRNAs with 4 or 5 exons. However, the number was still less than the number of mRNA exons. In terms of the transcription length, most of the mRNAs had lengths of >3000, especially the novel mRNAs, with a probability of over 70%. Most of the known lncRNAs had transcription lengths of <1000, and about 20% of the novel lncRNAs had transcription lengths of >3000. Novel lncRNAs are longer than known lncRNAs, but still shorter than protein coding mRNA. Then we analyzed the localization of mRNA and lncRNA, and found that novel mRNA was mainly concentrated on chromosome 3 (Figure 2d), while novel lncRNAs were mainly concentrated on the short and long arms of chromosome 2 (Figure 2e), which may have been determined by the number of genes on these two chromosomes.

We also analyzed the transcript types (Figure S1A). Of all the differentially expressed transcripts, 67.6% were known protein-coding RNAs, 13.1% were novel mRNAs, 9.4% were transposons, 4.5% were known and 1.5% novel lncRNAs. We classified these lncRNAs (Figure S1B), and more than half of the lncRNAs were lincRNAs (58.4%), while the remaining lncRNAs were intron lncRNA (25.4%), sense-lncRNA (9.5%), and antisense-lncRNA (6.7%). Then we performed a principal component analysis (PCA) on all mRNA and lncRNA transcripts (Figure S1C,D). The principal component analysis is based on the dimensionality reduction of all transcripts obtained. Finally, we extracted two components to replace all the transcription of the information. As can be seen from Figure S1, the PCA results of mRNA showed that the sample gaps within all groups were small. This shows that the sample difference within the group is small; however, the lncRNA results showed a larger sample gap in the male mutant. This may be because the expression level of lncRNA is not as stable as that of mRNA.

### 2.3. LncRNAs Expression Changes Significantly in ISWI-Mutated Background

**Figure 3:**
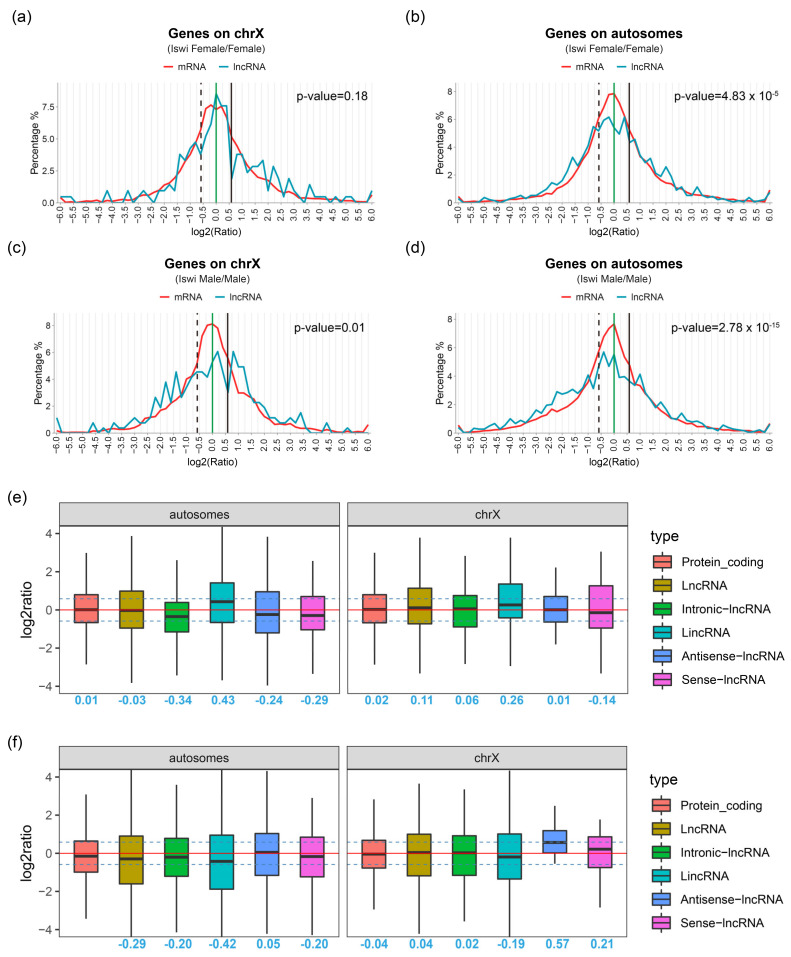
I Ratio distributions of transcripts expression in *ISWI* mutant compared with wild-type *Drosophila*. (**a**,**b**) Expression changes in transcripts on chrX (**a**) and on autosomes (**b**) in female. (**c**,**d**) Expression changes in transcripts on chrX (**c**) and on autosomes (**d**) in male. The x-axis is the logarithm base 2 of the ratio of the expression level of the transcript in the experimental group to the expression level of the transcript in the control group, which represents the relative expression level of the transcript; the ordinate is the distribution frequency of the transcript. The green vertical line represents log2 (Ratio) = 0.0, that is, Ratio = 1.0, which represents the theoretical value of dosage compensation; the black vertical line represents log2 (Ratio) = 0.58, indicating the positive dosage effect; the black dotted line shows log2 (Ratio) = −0.58, representing the inverse-dosage effect. The red line represents the frequency-relative expression distribution of the mRNA transcript, and the blue line represents the frequency-relative expression distribution of the lncRNA transcript. *p*-values are shown beside the graphs. (**e**,**f**) Box diagram of expression of different types of transcripts on autosomal and X chromosomes in female (**e**) and male (**f**). The x-axis is the transcript type and the ordinate are the log2 value of the ratio of *ISWI* mutant to wild type. The black horizontal line is the median of the box plot, the upper and lower quartiles of the box plot, and the vertical line is the upper and lower limits of the data. Medians are shown in blue number below the box plot for each group.

In order to explore the effect of *ISWI* mutation on the global gene expression of *Drosophila*, especially on lncRNA, we analyzed the gene expression of all transcripts and compared them with wild-type *Drosophila* to obtain the gene expression ratio distribution of *ISWI* mutant and wild-type *Drosophila* (Figure 3).

In mutant female *Drosophila*, there was no significant difference in the expression distribution of mRNA and lncRNA transcripts on the X chromosome. The highest peak appeared at the solid green line (Log2 (Ratio) = 0.0), where mRNA expression did not change significantly. The numbers of up-regulated and down-regulated transcripts on the X chromosome of female mutant flies were similar (Figure 3a). The peak of the mRNA transcripts located on autosomes was still at the solid green line, but the distribution of lncRNAs was significantly different from that of mRNA transcripts. LncRNAs near the solid green line decreased, and two peaks appeared on both sides, indicating that more lncRNAs had increased or decreased. In other words, lncRNA was modulated to a greater degree (Figure 3b). In male mutant *Drosophila*, the peak of mRNA transcripts located on the X chromosome appeared on the green solid line, while the lncRNA peak appeared on the right side of the green solid line (the expression of these lncRNAs was up-regulated). In terms of the total number of lncRNA transcripts, the numbers of up-regulated and down-regulated lncRNA transcripts were similar. There was an obvious peak near log2 = 1.0, where the expression of lncRNA was upregulated twice, which may have been related to the enlargement of the X chromosome in the *ISWI* mutant (Figure 3c). It has been demonstrated that transcription factors may cooperate with chromatin remodeling complexes to regulate transcription [28], and studies have shown that mutations in transcription factors disrupt dosage compensation in *Drosophila* [29]. Therefore, we independently analyzed the transcription factors on the X chromosome (Figure S2) and found that the transcription factors on the X chromosome of the male *Drosophila* were significantly enriched in the Log2 (Ratio) range of 0–1, indicating that these transcription factors had an obvious up-regulation trend in *ISWI* mutated background (Figure S2D). The results of the lncRNA transcripts in male *Drosophila* were different from those in mRNA described above. LncRNA transcripts had a higher peak on the left side of the green solid line (the transcripts expression decreased). In the *ISWI* mutated background, the mRNA and lncRNA transcripts on the male *Drosophila* autosomes were more downregulated (Figure 3d). This may be related to the inverse effect of up-regulated transcription factors on the X chromosome.

LncRNAs were classified into four types, and an overall expression analysis of the different types was conducted (Figure 3e,f). The mean expression levels of protein-coding transcripts and lncRNA transcripts in both autosomes and X chromosomes in female *Drosophila* did not change due to *ISWI* mutations (the median was around log2 = 0). Further analysis of different types of lncRNAs showed that the average expression levels of the other three lncRNAs in autosomes, except lincRNA, were down-regulated (the median was less than 0), while the median of lincRNA was 0.43 in Log2, indicating that the average expression levels were up-regulated (Figure 3e). On the autosomes, the average expression level of protein-encoded transcripts of mutant flies was close to that of wild types (the median was −0.15), while the average expression level of lncRNA decreased to a certain extent (the median was −0.29). Among the four types of lncRNA, the lincRNA showed the most obvious downward trend (the median was −0.42). The intronic-lncRNA and sense-lncRNA showed similar downward trend (both medians were −0.20), while antisense-lncRNA did not show this downregulation (the median was 0.05). The average expression level of overall lncRNA did not show a downward trend on the X chromosome (the median was 0.04). Although lincRNA still showed a decline (the median was −0.19), antisense-lncRNA showed an obvious up-regulation trend (the median was 0.57) (Figure 3f).

Our results show that after *ISWI* mutation, the change in transcriptional expression in male *Drosophila* is greater than that in female *Drosophila*. Compared with mRNA transcripts, lncRNA transcription rate is significantly higher in presence of ISWI mutation.

### 2.4. Differentially Expressed mRNAs and lncRNAs

We analyzed the differentially expressed mRNAs and lncRNAs of the *ISWI* mutant line and wild type line in males and females (Figure S3A,B). In ISWI female mutants (IF), there were 6964 differentially expressed mRNAs (3814 up-regulated and 3150 down-regulated), and in ISWI male mutants (IM), there were 7109 differentially expressed mRNAs (3238 up-regulated and 3881 down-regulated). There were 412 lncRNAs (218 up-regulated and 194 down-regulated) in female mutants and 426 lncRNAs (222 up-regulated and 204 down-regulated) in male mutants.

We performed a cluster heat map analysis on these mRNAs and lncRNAs (Figure S3C,D). Due to the large number of genes, some specific contents were difficult to observe, so they were generally used to display the overall differential expression trend. It can be seen that in the clustering heat map, female and male mutants are clustered together, indicating that changes in gene expression caused by *ISWI* mutations are greater than differences between genders.

**Figure 4:**
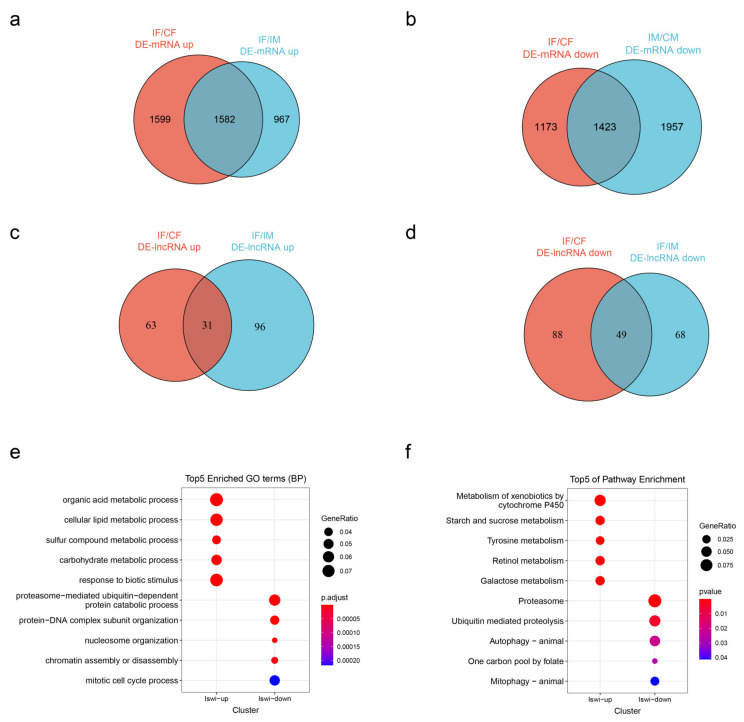
Differentially expressed known mRNAs and lncRNAs. (**a**,**b**) Venn diagrams show the number of up-regulated (**a**) and down-regulated (**b**) known-mRNAs in *ISWI* mutants/wild-type *Drosophila*. (**c**,**d**) Venn diagrams show the number of up-regulated (**c**) and down-regulated (**d**) known-lncRNAs in *ISWI* mutants/wild-type *Drosophila*. (**e**) GO enrichment analysis of differentially expressed known-mRNA. (**f**) KEGG pathway enrichment analysis of differentially expressed mRNA.

Subsequently, we analyzed known mRNAs and lncRNAs. We found 4148 up-regulated genes (1582 in both sexes) and 4553 down-regulated genes (1423 in both sexes) (Figure 4a,b). There are 190 up-regulated lncRNAs (31 in both sexes) (Figure 4c) and 205 down-regulated lncRNAs (49 in both sexes) (Figure 4d). The differentially expressed lncRNAs were analyzed in both sexes (Figure S4A). About 91 lncRNAs were differentially expressed in both sexes, and these lncRNAs were divided into four categories according to different expression trends (both up-regulated; both down-regulated; up-regulated in females and down-regulated in males; up-regulated in females and down-regulated in males) (Figure S4C). Of the 91 DE-lncRNAs, only 3 had known functions (Figure S4B).

We performed a GO functional enrichment analysis for differentially expressed mRNAs (Figure 4e). Metabolic processes, including organic acid metabolism, cellular lipid metabolism, sulfur compound metabolism and carbohydrate metabolism, were shown to be the main enrichment factors in up-regulated genes. Biological stimuli (detectable change (physical or chemical) in an organism’s environment that results in some functional activity) were also enriched in upregulated genes. The down-regulated genes were mainly concentrated in proteasome-mediated ubiquitin-dependent protein degradation, protein–DNA complex assembly, nucleosome assembly, chromosome assembly and disassembly, and the mitotic cell cycle.

KEGG pathway enrichment analysis was performed on genes corresponding to the differentially expressed mRNA transcripts. The figure shows the top 5 pathways with the most significant differential expression (Figure 4f). The up-regulated genes were mainly enriched in metabolic pathways, including the metabolism of exogenous substances through cytochrome P450, starch and sucrose, tyrosine, retinol and galactose. The down-regulated gene enrichment pathways were mainly related to the proteasome, ubiquitin-mediated proteolysis, autophagy, the folic acid carbon pool pathway and mitochondrial autophagy.

We used the protein–protein interaction (PPI) network to search for possible key genes in *ISWI* mutated fly lines. Proteins with high connectivity are more likely to be key actors in protein interaction networks. We performed the PPI analysis on up-regulated and down-regulated genes in *ISWI* mutant and wild-type *Drosophila*, respectively (Figure S5A,B), and identified the top 20 genes with connectivity (Figure S5C,D).

Proteins that directly interact with ISWI were screened from up-regulated and down-regulated interacting proteins (Figure S6A,C). Genes that encode these proteins were analyzed for functional enrichment. Among the upregulated genes, few were directly connected with ISWI, and their functions were mainly significantly enriched in nucleic acid metabolism regulation, chromatin remodeling, DNA repair, anterior and posterior end pattern specification, and digestive system development (Figure S6B). Among the down-regulated genes, many genes were found to directly interact with ISWI, and their functions were mainly concentrated in DNA conformation change, mitosis, RNA synthesis regulation, chromatin remodeling and central nervous system development (Figure S6D). Another important hub gene screening method is the MCODE software package analysis (Figure S6E–H). Many ISWI-related modules were identified in upregulated genes, whose functions were mainly enriched in the actin cytoskeleton, the DNA damage response, endocytosis, the meiotic cell cycle and maturation (Figure S6F). There were only nine genes in the down-regulated gene module, which were enriched in chromatin remodeling, cell proliferation regulation, histone modification, proteasome protein decomposition, and larval and pupal morphogenesis (Figure S6H).

### 2.5. Potential Interactors of DE-lncRNAs

**Figure 5:**
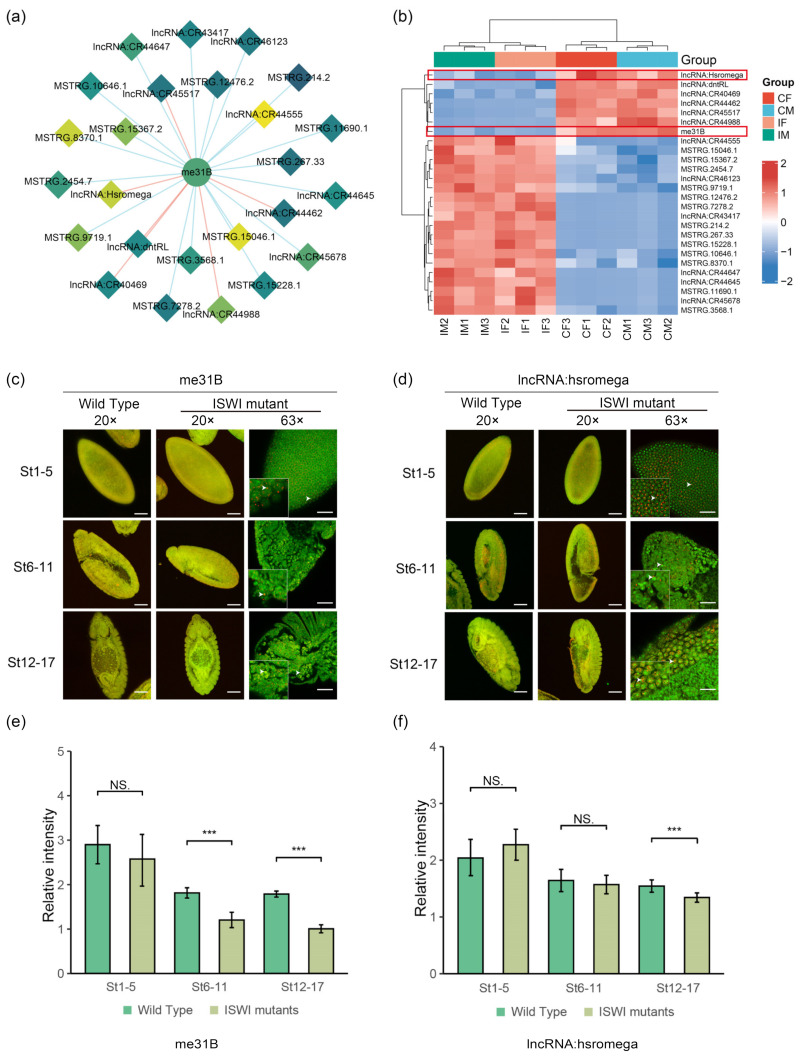
A possible interactor of DE-lncRNAs associated with ovarian development-*me31B*. (**a**) Network diagram of *me31B* and co-expressed lncRNAs. The diamond nodes represent lncRNAs, and the circle nodes represent mRNAs. The darker the color of node, the higher the degree of connection (the more co-expressed interactions). The red edge represents a positive correlation and the blue edge represents a negative correlation. (**b**) Clustering heat map of *me31B* and lncRNA expression levels in Figure 5a. (**c**,**d**) TSA-FISH results of *me31B* (**c**) and lncRNA: *hsromge* (**d**) in wild-type and mutant *Drosophila* embryos. 20×: Scale bars, 80 μm. The red pseudo-color is the signal from Probe and the green pseudo-color is the signal from nucleus. The genotype of the sample is shown in the horizontal axis above, and the development stage of the sample is shown in the left vertical axis. The red pseudo-color is the signal from Probe and the green pseudo-color is the signal from nucleus. RNA subcellular Location Patterns in mutant *Drosophila*. 63×: Scale bars, 30 μm. The white arrow head represents the subcellular localization of the probe signal. (**e**,**f**) Relative fluorescence intensity of *me31B* (**e**) and *hsromge* (**f**) signals in wild-type and mutant *Drosophila* embryos. The relative fluorescence intensity was calculated by the ratio of RNA signal to DAPI signal. The asterisk indicates *p* < 0.05 for the two-tailed Student’s *t*-test. NS. represents *p* value > 0.05 without significant difference, *** means abnormally significant difference, *p*-value was <0.001.

**Figure 6:**
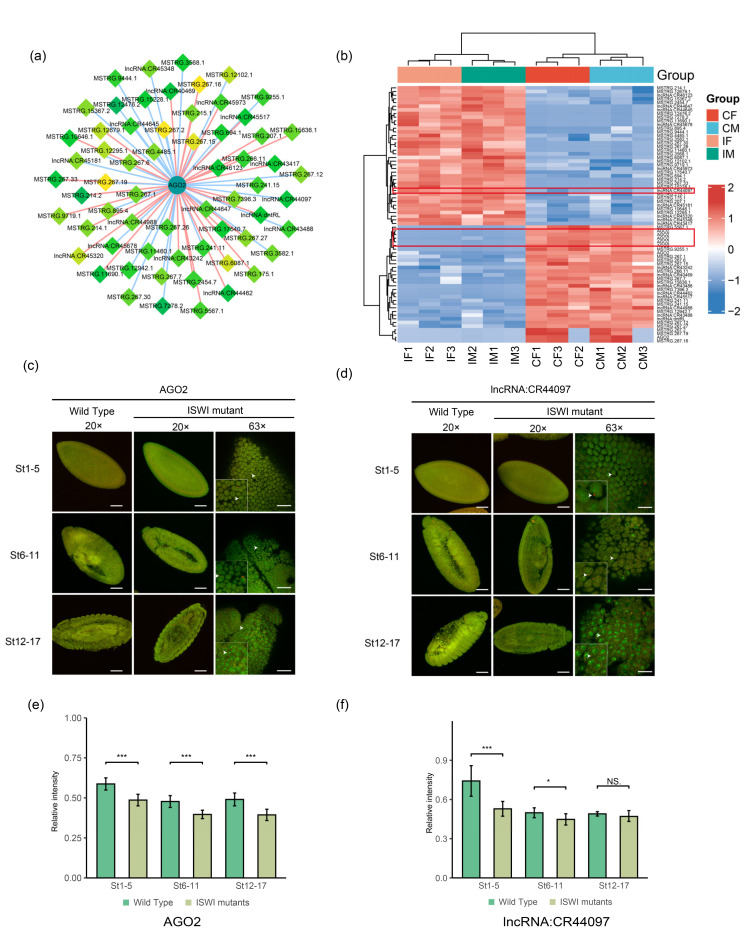
A possible interactor of DE-lncRNAs associated with transcription-*AGO2*. (**a**) Network diagram of *AGO2* and co-expressed lncRNAs. The diamond nodes represent lncRNAs, and the circle nodes represent mRNAs. The darker the color of node, the higher the degree of connection (the more co-expressed interactions). The red edge represents a positive correlation and the blue edge represents a negative correlation. (**b**) Clustering heat map of *AGO2* and lncRNA expression levels in Figure 6a. (**c**,**d**) TSA-FISH results of *AGO2* (**c**) and lncRNA:CR44097 (**d**) in wild-type and mutant *Drosophila* embryos. 20×: Scale bars, 80 μm. The red pseudo-color is the signal from Probe and the green pseudo-color is the signal from nucleus. The genotype of the sample is shown in the horizontal axis above, and the development stage of the sample is shown in the left vertical axis. The red pseudo-color is the signal from Probe and the green pseudo-color is the signal from nucleus. RNA subcellular Location Patterns in mutant *Drosophila*. 63× Scale bars, 30 μm. The white arrow head represents the subcellular localization of the probe signal. (**e**,**f**) Relative fluorescence intensity of *AGO2* (**e**) and CR44097 (**f**) signals in wild-type and mutant *Drosophila* embryos. The relative fluorescence intensity was calculated by the ratio of RNA signal to DAPI signal. The asterisk indicates *p* < 0.05 for the two-tailed student *t* test. NS. represents *p* value was >0.05 without significant difference. * means significant difference, *p* value was <0.05, *** means abnormally significant difference, *p*-value was <0.001.

**Figure 7:**
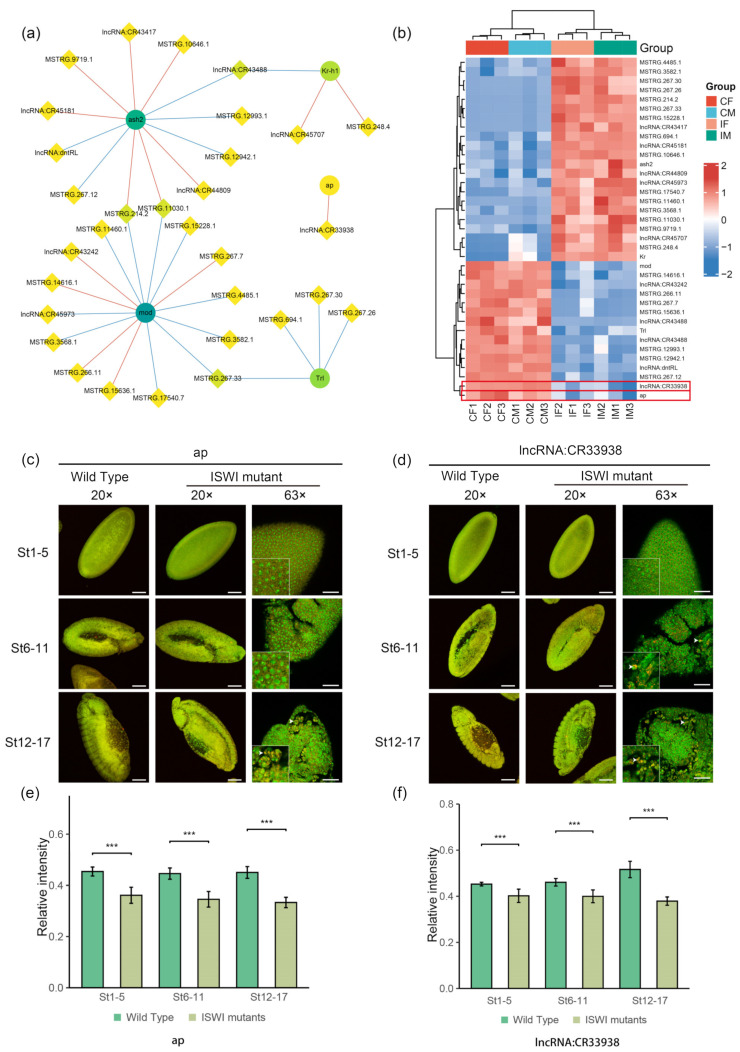
A possible interactor of DE-lncRNAs associated with trans-acting dosage-sensitive factors. (**a**) Network diagram of trans-acting dosage-sensitive factors and their co-expressed lncRNAs. The diamond nodes represent lncRNAs, and the circle nodes represent mRNAs. The darker the color of node, the higher the degree of connection (the more co-expressed interactions). The red edge represents a positive correlation and the blue edge represents a negative correlation. (**b**) Clustering heat map of trans-acting dosage-sensitive factors and lncRNA expression levels in Figure 7a. (**c**,**d**) TSA-FISH results of ap (**c**) and lncRNA:CR33938 (**d**) in wild-type and mutant *Drosophila* embryos. 20× Scale bars, 80 μm. The genotype of the sample is shown in the horizontal axis above, and the development stage of the sample is shown in the left vertical axis. The red pseudo-color is the signal from Probe and the green pseudo-color is the signal from nucleus. RNA subcellular Location Patterns in mutant *Drosophila*. 63× Scale bars, 30 μm. The white arrow head represents the subcellular localization of the probe signal. (**e**,**f**) Relative fluorescence intensity of ap (**e**) and CR33938 (**f**) signals in wild-type and mutant *Drosophila* embryos. The relative fluorescence intensity was calculated by the ratio of RNA signal to DAPI signal. The asterisk *** indicates *p* < 0.001 for the two-tailed student *t* test.

To search for possible interactor genes of DE-lncRNAs in *ISWI* mutants, we analyzed lncRNAs and their interactors. As a key factor in chromatin remodeling, ISWI plays an important role in many physiological activities. Differentially expressed genes (DEGs) were classified according to their functions, and seven genes with important functions and high connection degree were selected as ovarian development (Figure 5), transcription (Figure 6), and dosage-sensitive factors (Figure 7). We found that some known lncRNAs interacted with these genes through co-expression analysis. For example, lncRNA: *hsromge*, which is associated with nuclear spot formation, was shown to be co-expressed with E(bx) and *me31B*. LncRNA:CR44097 was found to be co-expressed with six selected genes but not *me31B*. LncRNA:CR40469 was also shown to be co-expressed with five genes. Two pairs of co-expressed lncRNAs and mRNAs with different functions were selected for subsequent embryo TSA-FISH experiments to explore their expression patterns and to speculate whether there is a possible regulatory relationship. The functions and co-expressed lncRNAs of the remaining screened genes are listed, and some lncRNAs were selected for further exploration (Figure S7) [30,31,32,33,34].

Among the ovarian-related genes, we selected *me31B*, which encodes an ATP-dependent RNA helicase and is a core component of various ribonucleic protein complexes (RNPs). It plays important roles in embryogenesis, oogenesis, neurogenesis and neurotransmission [35]. During oocyte development, *me31B* is involved in oocyte RNA localization and protein transport [36]. We analyzed DE-lncRNAs co-expressed with *me31B*, most of which were novel lncRNAs (name and function unknown) (Figure 5a). Among the known lncRNAs, only lncRNA: *hsromge* has a known function. It can generate multiple nuclear and cytoplasmic transcripts. Long nuclear transcripts bind to a variety of different ribonucleoproteins, and tissue cytoplasmic omega spots regulate a variety of cellular processes [37]. We performed a cluster analysis on *me31B* and its co-expressed lncRNAs (Figure 5b). *Me31B* was found to be downregulated in mutant *Drosophila*, but most co-expressed lncRNAs showed an increasing trend. We performed TSA-FISH on wild-type and mutant *Drosophila* embryos using *me31B* and lncRNA: *hsromge* RNA probes (see Table S1) to explore their expression and localization in embryos.

The embryonic TSA-FISH results showed that *me31B* was weakly expressed at all three stages. There was no obvious enrichment based on fluorescence quantification using the DAPI signal as a calibrator (Figure 5c) whereas lncRNA: *hsromge* was found to be significantly enriched (Figure 5d). During early embryonic development, *hsromge* was shown to be enriched in the embryo head. In the middle and late stage of development (St6-17), probe signals were found to be enriched in the amnioserosa (Figure 5d). In terms of subcellular localization, *me31B* was identified as having mainly nuclear and perinuclear localization, while the localization of *hsromge* varied according to the stage of embryo development. In the early stage, *hsromge* is located in the nucleus. In the middle and late stages of embryo development, localization occurs in and around the nucleus. This indicates that *me31B* and *hsromge* might co-locate to a certain extent (Figure 5c,d) [38,39].

In terms of the expression of RNA in embryos, *ISWI* mutations did not cause significant differences in the expression of *me31B* and *hsromge* in embryo development (St 1-5). However, with the continuous development of embryos, the expression of *me31B* decreased significantly after mutation. Downregulation of *hsromge* occurred in the late stages of embryonic development (St 12-17) (Figure 5e,f), which is consistent with our larval sequencing results (Red square in Figure 5b). This suggests that, upon *ISWI* mutation, there is a direct correlation in the expression levels of *hsromge* and *me31B*.

Among the transcription-related genes, the protein AGO2 with the highest connection degree was selected. *AGO2* is a crucial component of the RNA-induced silencing complex in siRNA-triggered RNA interference [40]. There are five different transcripts of *AGO2* in ISWI mutants. *Drosophila AGO2* contains an unusual amino-terminus with two types of imperfect glutamine-rich repeats (GRRs) of unknown function [40,41]. A large number of lncRNAs were found to be co-expressed with *AGO2*, and most of these lncRNAs were unknown (Figure 6a). In the cluster analysis of *AGO2* and lncRNAs, we found that all *AGO2* transcripts showed a down-regulation trend after mutation, and the numbers of up-regulated and down-regulated co-expressed lncRNAs were similar (Figure 6b). Among all known lncRNAs, lncRNA:CR44097 has high connection degree and is co-expressed with multiple important genes. CR44097 is located on chromosome 3R, but its molecular functions and biological processes are still unclear. We selected it for subsequent TSA-FISH studies along with *AGO2*.

As shown in Figure 6c, *AGO2* RNA probe showed no obvious enrichment trend during embryonic development. It was found to be weakly expressed at all stages of embryo development in both wild-type and mutant flies. The CR44097 results were similar, and no significant enrichment of RNA probe signals was found (Figure 6d) [38,39]. In subcellular localization, both *AGO2* and CR44097 probes showed obvious nuclear localization at the early stage of embryonic development (Figure 6c,d), with signals appearing near the edge of the nucleus. As embryos developed, the signals of the two RNA probes changed from nuclear localization (St 1-5) to perinuclear localization (St 12-17), indicating that co-localization of the two probes may exist at the subcellular level.

The probe signal showed that *ISWI* mutation resulted in decreased expression of *AGO2* at various stages of embryonic development (Figure 6e). At later stages of embryonic development (St 12-17), the result of this downregulation was similar to that of sequencing (Figure 6b). However, CR44097 had different results. The expression level of CR44097 was significantly reduced in *ISWI* mutant embryos during early embryonic development. This difference gradually diminished as the embryos continued to develop. In the final stages of development, there was no significant difference in the expression of CR44097 between mutated and wild-type embryos (Figure 6f). This trend could explain the up-regulation of CR44097 in mutant larvae (Figure 6b). The above results indicate that there may be certain interactions between *AGO2* and CR44097 (co-localization at the subcellular level). To some extent, this lncRNA may be involved in the regulation of AGO2 in *ISWI* mutation.

Studies of X and autosomal genes in embryos have shown that *ISWI* mutations can lead to overexpression of the X chromosome in male *Drosophila* at this early stage of development, suggesting that ISWI may be involved in the dosage compensation regulation of genes on the X chromosome together with the MSL complex [42]. We analyzed the expression levels of trans-acting dosage-sensitive factors in *ISWI* mutant RNA-seq data. Dosage-sensitive factors are single genes capable of a trans-acting effect, and 47 dosage-sensitive factors have been screened out by genetic techniques for trans-regulating *Drosophila* eye color [43]. In *ISWI* mutant *Drosophila*, we found significant differences in the expression of seven dosage-sensitive factors (*ash2*, *mod*, *Kr-h1*, *ap*, *Trl*, *Uba1,* and *Rb75D*). Among them, differentially expressed lncRNAs were found to be co-expressed with five dosage-sensitive factors (Figure 7a), but no lncRNAs were shown to be co-expressed with *Uba1* and *Rb75D*. We analyzed the differentially expressed lncRNAs co-expressed with these dosage-sensitive factors. Only a few lncRNAs were known, such as lncRNA:CR33938(co-expressed with *ap*) and lncRNA:CR44809(co-expressed with ash2). Cluster analysis was performed for these five dosage-sensitive factors and their co-expressed lncRNAs. The heat map showed that *ap, mod,* and *Trl* were down-regulated in the mutant, while *ash2* and *kr-h1* were up-regulated, and more co-expressed lncRNAs were up-regulated than down-regulated (Figure 7b).

The *ap* (*apterous*) gene encodes a transcription factor associated with dorsal recognition of *Drosophila* wing cells, muscle development, and nervous system development [44]. The TSA-FISH assay was performed on *ap* and its co-expressed lncRNA:CR33938(unknown function) in wild-type and *ISWI* mutant *Drosophila* embryos. No significant enrichment of *ap* and lncRNA:CR33938 was observed in *ISWI* mutant *Drosophila* embryos. Both RNA probes showed obvious nuclear localization in subcellular localization (Figure 7c,d) [38,39]. This suggests that *ap* and CR33938 may co-localize in the nucleus. It was seen that both *ap* and lncRNA:CR33938 were down-regulated to some extent after *ISWI* mutation (Figure 7e,f), which is consistent with the result obtained by sequencing (Figure 7a). Based on their common nuclear localization and common downregulation, data strongly suggest that the expression level of *ap* might be related to lncRNA:CR33938, meaning that *ap* may be a potential interactor of lncRNA:CR33938.

### 2.6. Differentially Expressed Transposons

**Figure 8:**
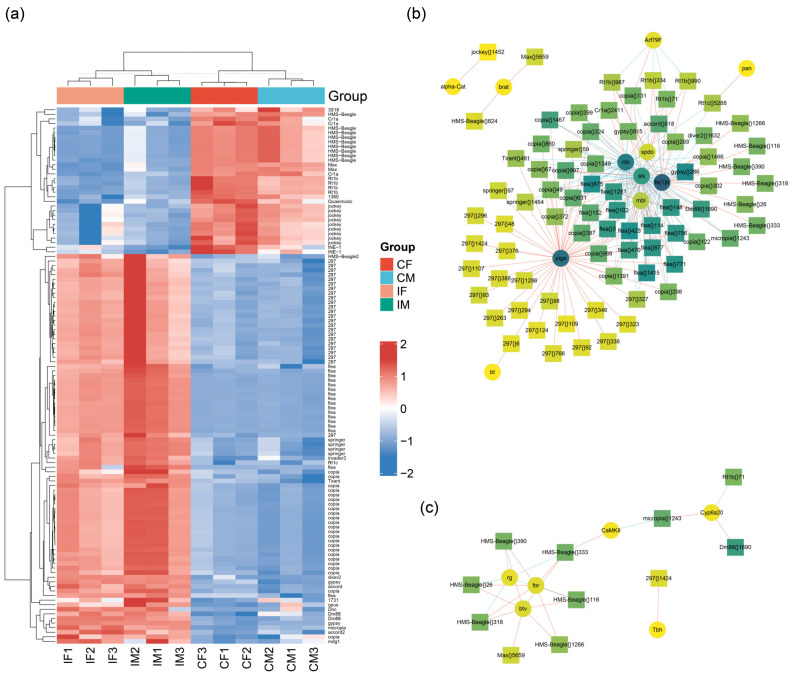
Differentially expressed transposons and co-expressed mRNAs. (**a**) Heat maps of differentially expressed transposon (baseMean > 500) expression level in *ISWI* mutant lines. Network of transposons associated with co-expressed mRNAs associated with growth and development (**b**) or chromatin remodeling (**c**). The pink line means positively correlated, and the blue line means negatively correlated.

Based on our research findings, lncRNA has a close relationship with transposons [14] and transposons play important roles in many physiological processes, such as heterochromatin formation [16]. Differentially expressed TE families were screened in the results of the transcriptional analysis. We conducted a cluster analysis for DE-TEs with baseMean > 500 (Figure 8a). As expected, the expression of some transposons in *ISWI* mutant *Drosophila* strains changed significantly, and the expression levels of the HMS-Beagle family, Rt1b family and jockey family TEs all showed significant down-regulation. However, more TE families showed up-regulated expression in mutant lines, such as the 297 family, flea family, and copia family.

We analyzed these TE families. The top 10 TEs were ranked according to the number of DE-TEs, their proportions in the whole family and their expression levels (baseMean) (Figure S8A–C). It was found that most of the top 10 TEs belong to the copia family. In terms of the absolute number and proportion of the TE family, copia ranked 3rd and 4th respectively, indicating that there are many highly expressed TEs in the copia family. In addition to copia, Burdock, flea, 297, and Tirant were among the top 10 TE families in terms of both quantity and proportion. We analyzed the location of TEs (Figure S8D) and found that most of the DE-TEs map on chromosome 2, which may be related to the number of transcripts on the chromosome.

We analyzed the differentially expressed genes co-expressed with differentially expressed TEs (baseMean > 500) and screened for genes related to *Drosophila* growth and development (Figure 8b). Other mRNAs were found to be associated with chromatin remodeling (Figure 8c). Two genes with high levels of expression and their co-expressed transposons were selected for the TSA-FISH experiment. We selected two TEs with different expression trends (MAX down-regulated in the mutant and 297 up-regulated in the mutant) from the DE-TEs (Figure 8a), and searched for their co-expressed genes (Figure 8b). Then we conducted embryonic TSA-FISH experiments on these two genes and their co-expressed transposons. The *Drosophila* gene *brat* encodes a tumor suppressor protein that, when mutated in neuroblasts, causes excessive proliferation of neuroblasts which, in turn, makes the brain significantly larger [45,46]. Another gene, *bent* (*bt*), encodes a large protein associated with the myosin filaments found in insect muscles that contribute to the stiffness of flight muscles. When *bt* is knocked down, the filaments that make up the *Drosophila* muscle are significantly reduced in length, resulting in a deficiency in the maintenance of the sarcomere lengths in adult muscles [47].

As shown in Figure S7A,B, the *brat* signal was significantly enriched in the embryo heads during embryonic development, and still existed in the *ISWI* mutant *Drosophila* embryos but not as obviously as in the wild-type embryos (Figure S9A). The transposon *MAX {}5659* was ubiquitously expressed throughout the embryos at all stages of embryonic development. There was no obvious enrichment (Figure S9B), and the subcellular localization of both was cytoplasmic localization (Figure S9A,B). The signal intensity of the RNA probe *brat* decreased due to *ISWI* mutation, which is consistent with the down-regulation trend shown in the larva sequencing results (Figure S9E). The relative fluorescence intensity of *MAX {}5659* in mutant *Drosophila* embryos was not significantly different from that in wild types (Figure S9F). In wild-type embryos, *bt* was found to be weakly expressed in early embryonic development without significant enrichment. However, it was significantly enriched in embryonic muscle tissue at the late stages (St 12-17). Surprisingly, this enrichment disappeared in the *ISWI* mutant embryos (Figure S9C), but there was an increase in fluorescence signal intensity (Figure S9G). This result is consistent with the up-regulation shown in the larva sequencing data. TE *297{}6*, co-expressed with *bt,* was widely expressed during the development of wild-type embryos, with no obvious location-enrichment at different stages (Figure S9D). In subcellular localization, both *bt* and *297* showed cytoplasmic localization (Figure S9C,D). In mutant embryos, the probe signal intensity of *297{}6* did not differ significantly from that of wild-type embryos (Figure S9H).

The above results show that both genes were significantly enriched in the embryos, and the enrichment of *bt* disappeared in the *ISWI* mutants. The signal intensities of RNA probes in late embryonic development were consistent with the results of larval sequencing, but the expression levels did not differ significantly in embryos due to *ISWI* mutation. With the development of embryos, the relative fluorescence intensity of the TE *MAX {}5659* probe showed a decreasing trend in the ratio of mutant to wild-type embryos, while that of the *297{}6* probe showed an increasing trend (Figure S9I). This trend may explain the differential expression of transposons in larval sequencing *(MAX {}5659* is down-regulated and *297{}6* is up-regulated).

## 3. Discussion

Chromatin is composed of nucleosomes, repeated units of histone octamers surrounded by approximately 147 bp of DNA. Nucleosomes constitute a barrier to DNA binding factors that reduce the accessibility of DNA sequences [48,49]. Therefore, in order for replication and transcription regulators to interact with DNA binding sites, the structure of chromatin, especially the location of nucleosomes, must be precisely and dynamically regulated [50]. Evidence suggests that chromatin folding or looping is also important for the regulation of enhancer and promoter interactions and for chromosome subdivision into discrete functional domains [51]. ISWI is an important chromatin remodeling factor involved in *Drosophila* transcription, chromatin remodeling, ovarian development, and other physiological activities. Mutations in the components of ISWI complexes, such as NURF [5] and ACF [3], will lead to growth and reproduction disorders in *Drosophila*. Studies have proved that the activity of ISWI is related to histone H4K16ac in vitro [4].

In this study, we obtained transcriptional sequencing data from *ISWI* mutant larvae and analyzed the characteristics and differential expression of mRNA and lncRNA in comparison with normal *Drosophila*. Our results show that the expression changes of lncRNAs following *ISWI* mutation are more obvious than those of mRNAs, and different types of lncRNAs were found to be regulated to different degrees. More interestingly, the expression levels of lncRNAs in males were shown to change more significantly than in females. In particular, the transcripts on the X chromosome appeared to undergo a certain degree of increase, which may be attributed to the abnormal enlargement of the X chromosome following *ISWI* mutation. Furthermore, it was found that ISWI might exhibit some functions related to genomic imbalance [52]. In addition, it was found that lncRNAs are more sensitive to genomic disorders in aneuploidy such as trisomy 21 syndrome (unpublished data from our lab). Thus, based on the significant differences in lncRNAs shown in this study, it is speculated that lncRNAs play an important role in the regulation of gene expression in chromatin remodeling processes.

LncRNAs have been extensively studied in many organisms [53]. LncRNAs participate in a variety of cellular processes, such as development, differentiation, and proliferation, and often contribute to the modulation of gene expression programs [9]. In addition to what we have mentioned above, lncRNA mainly affects gene expression at three levels: chromatin regulation, transcriptional regulation, and post-transcriptional regulation [10]. It also plays an important role in development [54]. In a high-throughput expression analysis of different mammalian tissues, researchers found that lncRNA may play a role in regulating cell fate determination [55]. The RNA-seq data from *Drosophila* also showed a large number of differentially expressed lncRNAs during development [56]. Studies in mice have proved that the interaction between lncRNA and PCR2 can remodel the chromatin structure [57]. In *Drosophila*, in addition to *roX1* and *roX2* mentioned above, there are some regulatory lncRNAs related to PcG and TrxG proteins [10]. Here a total of 384 known lncRNAs were analyzed in *ISWI* mutant flies, divided into lincRNA (58.4%), intronic lncRNA (25.4%), sense-lncRNA and antisense-lncRNA (9.5% and 6.7%). However, the functions of most lncRNAs have not been discovered yet; only the sequences are known at present, and relevant studies are very limited. In both male and female samples, three known functional lncRNAs (lncRNA:CR31781, lncRNA:CR43306 and lncRNA: *hsromge*) were analyzed from the 91 differentially expressed lncRNAs, and the functions of other lncRNAs need to be inferred from their co-expressed mRNA transcripts. However, among the lncRNAs with known functions, we only focused on *hsromge*, which is related to nuclear speckle formation [33]. We found that *hsromge* was significantly enriched in the amnioserosa of embryos, while at the subcellular level, we found that *hsromge* was mainly located in the nucleus at the early stage of embryogenesis. With the development of embryos, more perinuclear localization appeared. Although the mutation of *ISWI* did not lead to changes in lncRNA enrichment in embryos, the probe intensity was significantly reduced, and the expression level of the genes co-located with it also decreased. We speculated that *hsromge* might play an important role in the differential expression of genes in *ISWI* mutants. No co-expressed mRNAs were found for the other two lncRNAs, so it is possible that they play a limited regulatory role in the *ISWI* mutated background.

In *Drosophila* ovarian development, ISWI is necessary for the normal function of germline stem cells (GSC), as demonstrated in previous studies [58]. ISWI also plays an important role in transcription [5]. Studies in embryos have shown that ISWI may be related to dosage compensation [35] and may be an important mechanism to prevent the overactivation of the X chromosome in male *Drosophila* [59]. Therefore, we screened some important candidate genes through a GO analysis to assess three aspects: ovarian development, transcription, and trans-acting dosage-sensitive factors. Co-expressed lncRNAs of these genes were analyzed (absolute value of Pearson correlation coefficient greater than 0.95), and genes with high connectivity (more co-expressed lncRNAs) were screened out. Three genes were selected from different functional modules for subsequent analysis: *maternal expression at 31B* (*me31B*), which is related to ovarian development; transcription-related *Argonaute 2*(*AGO2*); and the trans-acting dosage-sensitive factor *Apterous* (*ap*). LncRNAs co-expressed with these genes were identified, and the embryo TSA-FISH assay was performed using RNA probes with better expected results. All results showed that the expression patterns of interactors at the last stage of embryonic development based on probe intensity were consistent with the RNA sequencing trend. The co-expression and co-localization results for these lncRNAs and mRNAs suggest that lncRNAs may play a role in the regulation of these mRNAs, but the specific regulatory mechanism involved needs to be further explored. At the same time, we also screened some other mRNAs and co-expressed lncRNAs. The interaction between them could be further verified by embryo experiments or immunoprecipitation experiments. This provides a new idea for exploring the possible functions and mechanisms of lncRNA.

Some lncRNAs have sequence similarity with transposable elements (TEs) [14], which can be roughly divided into two categories: DNA transposons and retrotransposons [60]. Most of the screened transposons were retrotransposons. DNA transposons move in DNA mainly by a cut-and-paste mechanism, while retrotransposons move via RNA by a copy-paste mechanism [61]. Due to the relationship between lncRNA and transposons, we also analyzed the data to explore the transposon expression existing in *ISWI* mutants, generated a differential expression heat map of transposons with high expression (baseMean > 500), and conducted a co-expression gene analysis of these transposons. Based on the differential expression in the mutants and the TSA-FISH results of the co-expressed genes, we selected two transposons that were up-regulated (*297{}6*) and down-regulated (*MAX {}5659*) in the mutants, and performed TSA-FISH experiments on the two candidate transposons and their co-expressed genes in embryos. There were no significant changes in the intensity of transposon RNA probes in mutant and wild-type embryos, but with the development of embryos, the change trend was consistent with the larval sequencing results. This may be due to the low expression of transposons in embryos, meaning that the changes in probe signal intensity were not as significant as those of mRNAs and lncRNAs. Currently, there have only been some studies on the role of transposons in chromatin remodeling in plants, and the authors of this study suggest that transposons could affect epigenetic regulation, including chromatin remodeling [59], and there have been very limited studies conducted in this area in fruit flies. Therefore, the specific functional mechanism of transposons in chromatin remodeling needs to be further studied to provide a scientific basis for understanding the global expression regulation related to chromatin remodeling.

## 4. Materials and Methods

### 4.1. Drosophila Stocks and Crosses

Flies were cultured on cornmeal dextrose medium at 25 °C. The *ISWI* mutant *Drosophila* strains used in this experiment were provided by the laboratory of James Birchler, Department of Biology, University of Missouri, USA. Their genotypes are: yw; ISWI1/CyO·GFP andyw; ISWI2/CyO·GFP. Yellow and white are two genes, and yellow controls the body color while the white controls the eye color on chromosome X. ISWI1 and ISWI2 are genes on chromosome 2. ISWI1/ISWI2 third instar larvae were collected based on non-GFP signals from the crosses.

### 4.2. Immunostaining of Chromosomes

Polytene chromosomes from the third instar larvae were dissected, fixed, and processed for antibody staining according to reference [62].

### 4.3. Embryo TSA-FISH

Designed primers were used to amplify the DNA fragments of the genes to be detected. The primers containing flanking T3 and T7 promoter elements are listed in Table S1. Detailed experimental steps are as described by Zhang S, et al. [62], and Le’cuyer et al. [63].

The embryos were divided into three groups according to the development stage. Fluorescence photos of embryos were taken with the same parameters (ZEISS Inverted Fluorescence Microscopy Observer Z1, RNA probe exposure time: 9.2 s, DAPI exposure time: 2.9 s). Fluorescence quantification was performed (Image J 1.8.0) (National Institute of Mental Health, Bethesda, MD, USA). on embryos at different development stages, and the ratio of RNA signal (red) to DAPI signal (green) was calculated. The description of embryonic and subcellular localization of signals refers to the Fly-Fish (http://fly-fish.ccbr.utoronto.ca/ accessed on 20 April 2022) and literatures [38,39].

### 4.4. RNA Extraction and Sequencing

Male and female samples of the third instar larvae of wild-type and mutant *Drosophila* were collected. There are three replicates in each group with about 20 larvae in each replicate. First, the sequencing library was constructed by removing ribosomal RNA to obtain a transcriptome containing protein-coding genes and non-coding genes. Then, the RNA library is subject to quality detection and quantification. Finally, the sequencing was performed using Illumina NovaSeq 6000 sequencers with the paired-end 150 bp protocol.

### 4.5. RNA-Seq Reads Mapping and Transcriptome Assembly

In order to have high-quality data for subsequent analysis steps, FastQC (version 0.11.9) (the Babraham Institute, Cambridge, UK) was used to assess the quality of the original reads, Trimmomatic (version 0.39) (THE USADEL LAB, Aachen, Germany) was used to filter the raw data [64]. The lower quality reads were deleted, and the remaining high-quality clean reads were mapped to the *Drosophila* genome (version 104) in conjunction with the corresponding annotation with the default parameters by the spliced reads aligner HISAT2 (version 2.2.1) (Lyda Hill Department of Bioinformatics, Dallas, TX, USA) [65]. Subsequently, SAMtools (version 1.12) (Wellcome Trust Sanger Institute, Cambridge, UK) was used to convert the alignment files from SAM to BAM format. Transcripts were assembled and quantitated using StringTie (version 2.1.5) (The Center for Computational Biology, Baltimore, MD, USA) [66]. gffcompare (version 0.11.2) (The Center for Computational Biology, Baltimore, MD, USA) was used to compare the combined GTF files with reference annotation.

### 4.6. Transcripts Filtering and Prediction

The principal pipeline employed for novel lncRNA identification was as follows “Transcripts Filtering and Prediction” (Figure 2a): (1) Single exon transcripts and transcripts less than 200 nt in length were deleted from the predicted transcripts [67]; (2) collect the positional information of the exons of the remaining transcripts, extract the genomic sequence of the corresponding exon, and fuse into a complete transcript sequence; (3) use CPC2 to assess the protein coding potential of complete transcript sequences, retaining transcripts that cannot encode proteins based on protein coding potential [68]; (4) eliminate transcripts containing any known protein coding domain, using Transeq2 in EMBOSS (version 6.6.0) (EMBOSS Credits, London, UK) to translate transcripts sequences into six possible protein domains and if the corresponding transcripts had a significant hit in the Pfam database (E-value < 1 × 10^−5^), they were discarded [69]. Using DIAMOND (version 0.9.24) (Benjamin Buchfink at the Drost lab, Tübingen, Germany) to filter out transcripts which is similar to a known protein with an E-value < 1 × 10^−5^ in the NR and the UniRef90 database [70].

### 4.7. Comparisons between lncRNAs and mRNA Transcripts

After transcripts quality control and alignment, 27,656 known mRNA and 1859 known lncRNA transcripts were analyzed. In addition to these transcripts, through filtering and prediction, we obtained 5369 novel mRNA and 600 lncRNA transcripts. We then identified all transcripts of mRNA and lncRNA in terms of exon number, transcript length, and expression level. Finally, we compared the different characteristics and localization of lncRNA and protein-coding transcripts in terms of exon number and transcript length [67].

### 4.8. Ratio Distribution

Read counts were normalized in DESeq2 and averaged across biological replicates. The ratio of each transcript was calculated by dividing the experimental value by the control value, and the logarithm was taken to make the ratio distributions. The plots were generated using ggplot2 package (version 3.3.5) in the R program (Lucent Technologies, NJ, USA).

### 4.9. Differential Expression Analysis

To analyze the differential expression of these mRNA and lncRNA, DEseq2 (version1.32.0) (Michael Love, Gillings School of Global Public Health, Chapel Hill, NC, USA) was used to analyze the data. ClusterProfiler (version 4.0.2) (Guangchuang Yu, Southern Medical University, Guangdong, China) was used for enrichment analysis. The GO annotations were provided by org.Dm.eg.db (version 3.13.0) (Bioconductor). Protein–protein interaction network analysis was conducted using online tool STRING 11.0 (https://string-db.org/ accessed on 23 March 2022) [71]. The networks were further processed by Cytoscape (version 3.7.1) (Institute for Systems Biology, Seattle, WA, USA) [72]. Heatmaps were generated using ComplexHeatmap (version 2.8.0) (Zuguang Gu, German Cancer Research Center, Heidelberg, Germany).

### 4.10. Potential Reactors of DE-lncRNA

In order to find the potential reactors of lncRNA, we calculated the Pearson correlation coefficient between all differentially expressed lncRNA and differentially expressed mRNA. LncRNA-mRNA pairs with correlation coefficients greater than 0.95 and *p*-values less than 0.05 were considered as candidates.

## Data Availability

The sequencing data has been deposited in the Gene Expression Omnibus (GEO) database (https://www.ncbi.nlm.nih.gov/geo/, accession no. GSE201291, accessed on 23 April 2022).

## Supplementary Materials

The following supporting information can be downloaded at: https://www.mdpi.com/article/10.3390/ijms23116247/s1.

## References

[1] Tsukiyama T., Danial C., Tamkun J., Wu C. (1995). ISWI, a member of the SWI2/SNF2 ATPase family, encodes the 140 kDa subunit of the nucleosome remodeling factor. Cell.

[2] Li Y., Gong H., Wang P., Zhu Y., Peng H., Cui Y., Li H., Liu J., Wang Z. (2021). The emerging role of ISWI chromatin remodeling complexes in cancer. J. Exp. Clin. Cancer Res..

[3] Borner K., Jain D., Vazquez-Pianzola P., Vengadasalam S., Steffen N., Fyodorov D.V., Tomancak P., Konev A., Suter B., Becker P.B. (2016). A role for tuned levels of nucleosome remodeler subunit ACF1 during *Drosophila* oogenesis. Dev. Biol..

[4] Klinker H., Mueller-Planitz F., Yang R.L., Forne I., Liu C.F., Nordenskiold L., Becker P.B. (2014). ISWI Remodelling of Physiological Chromatin Fibres Acetylated at Lysine 16 of Histone H4. PLoS ONE.

[5] Mizuguchi G., Tsukiyama T., Wisniewski J., Wu C. (1997). Role of nucleosome remodeling factor NURF in transcriptional activation of chromatin. Mol. Cell.

[6] Mizutani R., Wakamatsu A., Tanaka N., Yoshida H., Tochigi N., Suzuki Y., Oonishi T., Tani H., Tano K., Ijiri K. (2012). Identification and characterization of novel genotoxic stress-inducible nuclear long noncoding RNAs in mammalian cells. PLoS ONE.

[7] Li T., Mo X., Fu L., Xiao B., Guo J. (2016). Molecular mechanisms of long noncoding RNAs on gastric cancer. Oncotarget.

[8] Scheuermann J.C., Boyer L.A. (2013). Getting to the heart of the matter: Long non-coding RNAs in cardiac development and disease. EMBO J..

[9] Statello L., Guo C.J., Chen L.L., Huarte M. (2021). Gene regulation by long non-coding RNAs and its biological functions. Nat. Rev. Mol. Cell Biol..

[10] Camilleri-Robles C., Amador R., Klein C.C., Guigo R., Corominas M., Ruiz-Romero M. (2022). Genomic and functional conservation of lncRNAs: Lessons from flies. Mamm. Genome.

[11] Yang F., Deng X., Ma W., Berletch J.B., Rabaia N., Wei G., Moore J.M., Filippova G.N., Xu J., Liu Y. (2015). The lncRNA Firre anchors the inactive X chromosome to the nucleolus by binding CTCF and maintains H3K27me3 methylation. Genome Biol..

[12] Seila A.C., Calabrese J.M., Levine S.S., Yeo G.W., Rahl P.B., Flynn R.A., Young R.A., Sharp P.A. (2008). Divergent transcription from active promoters. Science.

[13] Hartford CCR L.A. (2020). When Long Noncoding Becomes Protein Coding. Mol. Cell Biol..

[14] Meller V.H., Rattner B.P. (2002). The roX genes encode redundant male-specific lethal transcripts required for targeting of the MSL complex. EMBO J..

[15] Lv Y., Hu F., Zhou Y., Wu F., Gaut B.S. (2019). Maize transposable elements contribute to long non-coding RNAs that are regulatory hubs for abiotic stress response. BMC Genom..

[16] Marsano R.M., Dimitri P. (2022). Constitutive Heterochromatin in Eukaryotic Genomes: A Mine of Transposable Elements. Cells.

[17] Percharde M., Sultana T., Ramalho-Santos M. (2020). What Doesn’t Kill You Makes You Stronger: Transposons as Dual Players in Chromatin Regulation and Genomic Variation. Bioessays.

[18] McCullers T.J., Steiniger M. (2017). Transposable elements in *Drosophila*. Mob. Genet. Elem..

[19] SanMiguel P., Tikhonov A., Jin Y.K., Motchoulskaia N., Zakharov D., Melake-Berhan A., Springer P.S., Edwards K.J., Lee M., Avramova Z. (1996). Nested retrotransposons in the intergenic regions of the maize genome. Science.

[20] Lander E.S., Linton L.M., Birren B., Nusbaum C., Zody M.C., Baldwin J., Devon K., Dewar K., Doyle M., FitzHugh W. (2001). Initial sequencing and analysis of the human genome. Nature.

[21] Tenaillon M.I., Hufford M.B., Gaut B.S., Ross-Ibarra J. (2011). Genome size and transposable element content as determined by high-throughput sequencing in maize and *Zea luxurians*. Genome Biol. Evol..

[22] Barron M.G., Fiston-Lavier A.S., Petrov D.A., Gonzalez J. (2014). Population genomics of transposable elements in *Drosophila*. Annu. Rev. Genet..

[23] Lannoy N., Hermans C. (2016). Principles of genetic variations and molecular diseases: Applications in hemophilia A. Crit. Rev. Oncol. Hematol..

[24] Pedro D.L.F., Lorenzetti A.P.R., Domingues D.S., Paschoal A.R. (2018). PlaNC-TE: A comprehensive knowledgebase of non-coding RNAs and transposable elements in plants. Database.

[25] Moschall R., Gaik M., Medenbach J. (2017). Promiscuity in post-transcriptional control of gene expression: *Drosophila* sex-lethal and its regulatory partnerships. FEBS Lett..

[26] Haussmann I.U., Bodi Z., Sanchez-Moran E., Mongan N.P., Archer N., Fray R.G., Soller M. (2016). m(6)A potentiates Sxl alternative pre-mRNA splicing for robust *Drosophila* sex determination. Nature.

[27] Corona D.F., Clapier C.R., Becker P.B., Tamkun J.W. (2002). Modulation of ISWI function by site-specific histone acetylation. EMBO Rep..

[28] Judd J., Duarte F.M., Lis J.T. (2021). Pioneer-like factor GAF cooperates with PBAP (SWI/SNF) and NURF (ISWI) to regulate transcription. Genes Dev..

[29] Prabhakaran M., Kelley R.L. (2012). Mutations in the transcription elongation factor SPT5 disrupt a reporter for dosage compensation in Drosophila. PLoS Genet..

[30] Espinas M.L., Canudas S., Fanti L., Pimpinelli S., Casanova J., Azorin F. (2000). The GAGA factor of *Drosophila* interacts with SAP18, a Sin3-associated polypeptide. EMBO Rep..

[31] Shaffer C.D., Stephens G.E., Thompson B.A., Funches L., Bernat J.A., Craig C.A., Elgin S.C. (2002). Heterochromatin protein 2 (HP2), a partner of HP1 in *Drosophila* heterochromatin. Proc. Natl. Acad. Sci. USA.

[32] Sharp K.A., Khoury M.J., Wirtz-Peitz F., Bilder D. (2021). Evidence for a nuclear role for *Drosophila* Dlg as a regulator of the NURF complex. Mol. Biol. Cell.

[33] Erickson J.W. (2016). Primary Sex Determination in *Drosophila* melanogaster Does Not Rely on the Male-Specific Lethal Complex. Genetics.

[34] Slack C., Alic N., Foley A., Cabecinha M., Hoddinott M.P., Partridge L. (2015). The Ras-Erk-ETS-Signaling Pathway Is a Drug Target for Longevity. Cell.

[35] Lee J., Yoo E., Lee H., Park K., Hur J.H., Lim C. (2017). LSM12 and *ME31B*/DDX6 Define Distinct Modes of Posttranscriptional Regulation by ATAXIN-2 Protein Complex in *Drosophila* Circadian Pacemaker Neurons. Mol. Cell.

[36] Nakamura A., Amikura R., Hanyu K., Kobayashi S. (2001). *Me31B* silences translation of oocyte-localizing RNAs through the formation of cytoplasmic RNP complex during *Drosophila* oogenesis. Development.

[37] Ray M., Acharya S., Shambhavi S., Lakhotia S.C. (2019). Over-expression of Hsp83 in grossly depleted *hsromge* lncRNA background causes synthetic lethality and l(2)gl phenocopy in *Drosophila*. J. Biosci..

[38] Lécuyer E., Yoshida H., Parthasarathy N., Alm C., Babak T., Cerovina T., Hughes T.R., Tomancak P., Krause H.M. (2007). Global analysis of mRNA localization reveals a prominent role in organizing cellular architecture and function. Cell.

[39] Wilk R., Hu J., Blotsky D., Krause H.M. (2016). Diverse and pervasive subcellular distributions for both coding and long noncoding RNAs. Genes Dev..

[40] Meyer W.J., Schreiber S., Guo Y., Volkmann T., Welte M.A., Muller H.A. (2006). Overlapping functions of argonaute proteins in patterning and morphogenesis of *Drosophila* embryos. PLoS Genet..

[41] Hammond S.M., Boettcher S., Caudy A.A., Kobayashi R., Hannon G.J. (2001). Argonaute2, a link between genetic and biochemical analyses of RNAi. Science.

[42] Bhadra M.P., Bhadra U., Kundu J., Birchler J.A. (2005). Gene expression analysis of the function of the male-specific lethal complex in *Drosophila*. Genetics.

[43] Xie W., Birchler J.A. (2012). Identification of Inverse Regulator-a (Inr-a) as Synonymous with Pre-mRNA Cleavage Complex II Protein (Pcf11) in *Drosophila*. G3.

[44] Yoo B., Kim H.Y., Chen X., Shen W., Jang J.S., Stein S.N., Cormier O., Pereira L., Shih C.R.Y., Krieger C. (2021). 20-hydroxyecdysone (20E) signaling regulates amnioserosa morphogenesis during *Drosophila* dorsal closure: EcR modulates gene expression in a complex with the AP-1 subunit, Jun. Biol. Open.

[45] Keegan S.E., Hughes S.C. (2021). Role of nuclear-cytoplasmic protein localization during *Drosophila* neuroblast development. Genome.

[46] Arama E., Dickman D., Kimchie Z., Shearn A., Lev Z. (2000). Mutations in the beta-propeller domain of the *Drosophila* brain tumor (brat) protein induce neoplasm in the larval brain. Oncogene.

[47] Perkins A.D., Tanentzapf G. (2014). An ongoing role for structural sarcomeric components in maintaining *Drosophila* melanogaster muscle function and structure. PLoS ONE.

[48] Li D., Liu J., Liu W., Li G., Yang Z., Qin P., Xu L. (2017). The ISWI remodeler in plants: Protein complexes, biochemical functions, and developmental roles. Chromosoma.

[49] Yaniv M. (2014). Chromatin remodeling: From transcription to cancer. Cancer Genet..

[50] Luger K., Mader A.W., Richmond R.K., Sargent D.F., Richmond T.J. (1997). Crystal structure of the nucleosome core particle at 2.8 A resolution. Nature.

[51] Misteli T. (2007). Beyond the sequence: Cellular organization of genome function. Cell.

[52] Clapier C.R. (2021). Sophisticated Conversations between Chromatin and Chromatin Remodelers, and Dissonances in Cancer. Int. J. Mol. Sci..

[53] Jandura A., Krause H.M. (2017). The New RNA World: Growing Evidence for Long Noncoding RNA Functionality. Trends Genet..

[54] Grote P., Wittler L., Hendrix D., Koch F., Währisch S., Beisaw A., Macura K., Bläss G., Kellis M., Werber M. (2013). The tissue-specific lncRNA Fendrr is an essential regulator of heart and body wall development in the mouse. Dev. Cell.

[55] Constanty F., Shkumatava A. (2021). lncRNAs in development and differentiation: From sequence motifs to functional characterization. Development.

[56] Li K., Tian Y., Yuan Y., Fan X., Yang M., He Z., Yang D. (2019). Insights into the Functions of LncRNAs in *Drosophila*. Int. J. Mol. Sci..

[57] Zhao W., Geng D., Li S., Chen Z., Sun M. (2018). LncRNA HOTAIR influences cell growth, migration, invasion, and apoptosis via the miR-20a-5p/HMGA2 axis in breast cancer. Cancer Med..

[58] Drummond-Barbosa D. (2019). Local and Physiological Control of Germline Stem Cell Lineages in *Drosophila* melanogaster. Genetics.

[59] Birchler J.A. (2016). Parallel Universes for Models of X Chromosome Dosage Compensation in *Drosophila*: A Review. Cytogenet. Genome Res..

[60] Finnegan D.J. (1989). Eukaryotic transposable elements and genome evolution. Trends Genet..

[61] Pradhan R.K., Ramakrishna W. (2022). Transposons: Unexpected players in cancer. Gene.

[62] Zhang S., Qi H., Huang C., Yuan L., Zhang L., Wang R., Tian Y., Sun L. (2021). Interaction of Male Specific Lethal complex and genomic imbalance on global gene expression in *Drosophila*. Sci. Rep..

[63] Lecuyer E., Parthasarathy N., Krause H.M. (2008). Fluorescent in situ hybridization protocols in *Drosophila* embryos and tissues. Methods Mol. Biol..

[64] Xiao H., Yuan Z., Guo D., Hou B., Yin C., Zhang W., Li F. (2015). Genome-wide identification of long noncoding RNA genes and their potential association with fecundity and virulence in rice brown planthopper, *Nilaparvata lugens*. BMC Genom..

[65] Pertea M., Kim D., Pertea G.M., Leek J.T., Salzberg S.L. (2016). Transcript-level expression analysis of RNA-seq experiments with HISAT, StringTie and Ballgown. Nat. Protoc..

[66] Pertea M., Pertea G.M., Antonescu C.M., Chang T.C., Mendell J.T., Salzberg S.L. (2015). StringTie enables improved reconstruction of a transcriptome from RNA-seq reads. Nat. Biotechnol..

[67] Chen L., Shi G., Chen G., Li J., Li M., Zou C., Fang C., Li C. (2019). Transcriptome Analysis Suggests the Roles of Long Intergenic Non-coding RNAs in the Growth Performance of Weaned Piglets. Front. Genet..

[68] Kong L., Zhang Y., Ye Z.Q., Liu X.Q., Zhao S.Q., Wei L., Gao G. (2007). CPC: Assess the protein-coding potential of transcripts using sequence features and support vector machine. Nucleic Acids Res..

[69] Finn R.D., Clements J., Arndt W., Miller B.L., Wheeler T.J., Schreiber F., Bateman A., Eddy S.R. (2015). HMMER web server: 2015 update. Nucleic Acids Res..

[70] Pirooznia M., Perkins E.J., Deng Y. (2008). Batch Blast Extractor: An automated blastx parser application. BMC Genom..

[71] Szklarczyk D., Gable A.L., Lyon D., Junge A., Wyder S., Huerta-Cepas J., Simonovic M., Doncheva N.T., Morris J.H., Bork P. (2019). STRING v11: Protein-protein association networks with increased coverage, supporting functional discovery in genome-wide experimental datasets. Nucleic Acids Res..

[72] Shannon P., Markiel A., Ozier O., Baliga N.S., Wang J.T., Ramage D., Amin N., Schwikowski B., Ideker T. (2003). Cytoscape: A software environment for integrated models of biomolecular interaction networks. Genome Res..

