# Supplementary material for: Genome-Wide Identification of Long Noncoding RNA and Their Potential Interactors in ISWI Mutants
Source: Int J Mol Sci. 2022 Jun 2;23(11):6247. doi: 10.3390/ijms23116247 (PMC9181106; doi:10.3390/ijms23116247)
Supplement: Supplementary file 1 [file ijms-23-06247-s001.zip › ijms-1723864-supplementary.pdf]

Supplementary Information

Genome-wide identification of long noncoding RNA and their potential interactors in ISWI mutants

Ludan Zhang [1, †], Shuai Zhang[1, †], Ruixue Wang[1,] and Lin Sun[1,] *.

1 Beijing Key Laboratory of Gene Resource and Molecular Development, College of Life Sciences, Beijing Normal University, Beijing 100875, China

* Author to whom correspondence should be addressed.

† L.Z., S.Z. and R.W. contributed equally to this work.

Table S1 Primer used in TSA-FISH

Transcript type	Transcript name	5′ sequence	3′ sequence
lncRNA	CR33938	CCTTGTTGGCTCATTCAGTG	GCTCTTAGTTCTTGGACGGA
	Hsromega	CAAACCCCCTGGAAAAGATG	GTATGGGGAAGCAAAACTGG
	CR44097	CACTGTATGATGGCCTTGAG	GAGTAATCGGGTTGTTTGGG
mRNA	brat	TCTATCAGACTCTCCGCTCA	GGCGTACTTGAACTCATTCG
	bt	GCCTGGGGATAAGTGTAATG	GTAAGACCTGTAACATCGGC
	ap	CAGTCACCTCCGACATCATA	GGGGAGTCAGGCTAATATGG
	me31B	ACGAGATCAACCTGATGGAG	CTCACCCAAATGACCGAAAC
	AGO2	GTGACATACAGACCGTTCAC	GATAGGCGTTACGGTACTCT
Transposon	297{}6	CCTCCTTTGAACATCACTCC	GTTGGAGTCTGGGTAAGGAA
	Max{}5659	TACGAGTGAAGAGATCCGAG	GGATAGCCTTCGTGGAAAAG

The sequences of promoters connected to the flanks of the primers are not listed in the table. The forward primers connect T3 promoter: TGTTGGGAAATCACTCCCAATTAA. The reverse primers connect T7 promoter: GTAATACGACTCACTATAGGGAGACCAC.

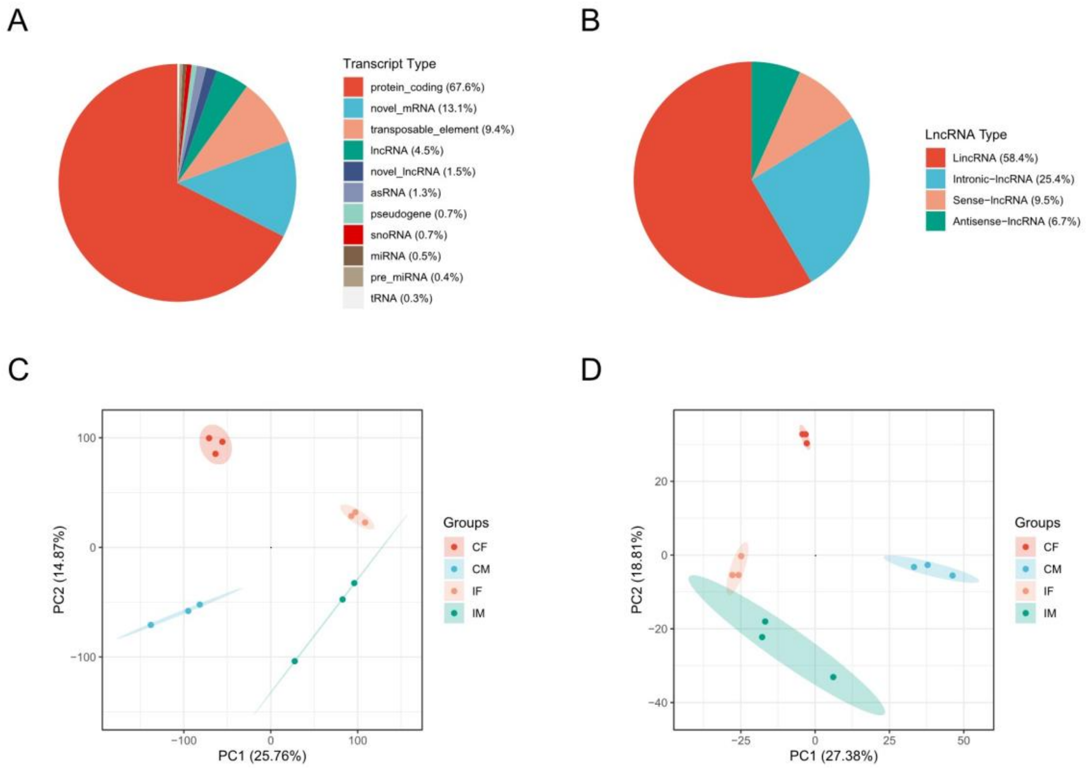

Figure S1 Overview of all transcripts of four kinds of samples. (A) Transcript type of all samples. (B)LncRNA types of green part ofFigureS1(A). (C.D) Principal component analysis (PCA) plots show mRNA(C) and lncRNA(D) in mutant samples and controls. The circle represents a 95% confidence interval. CF, wildtype female control; CM, wildtype male control; IF, ISWI mutant female; IM, ISWI mutant male.

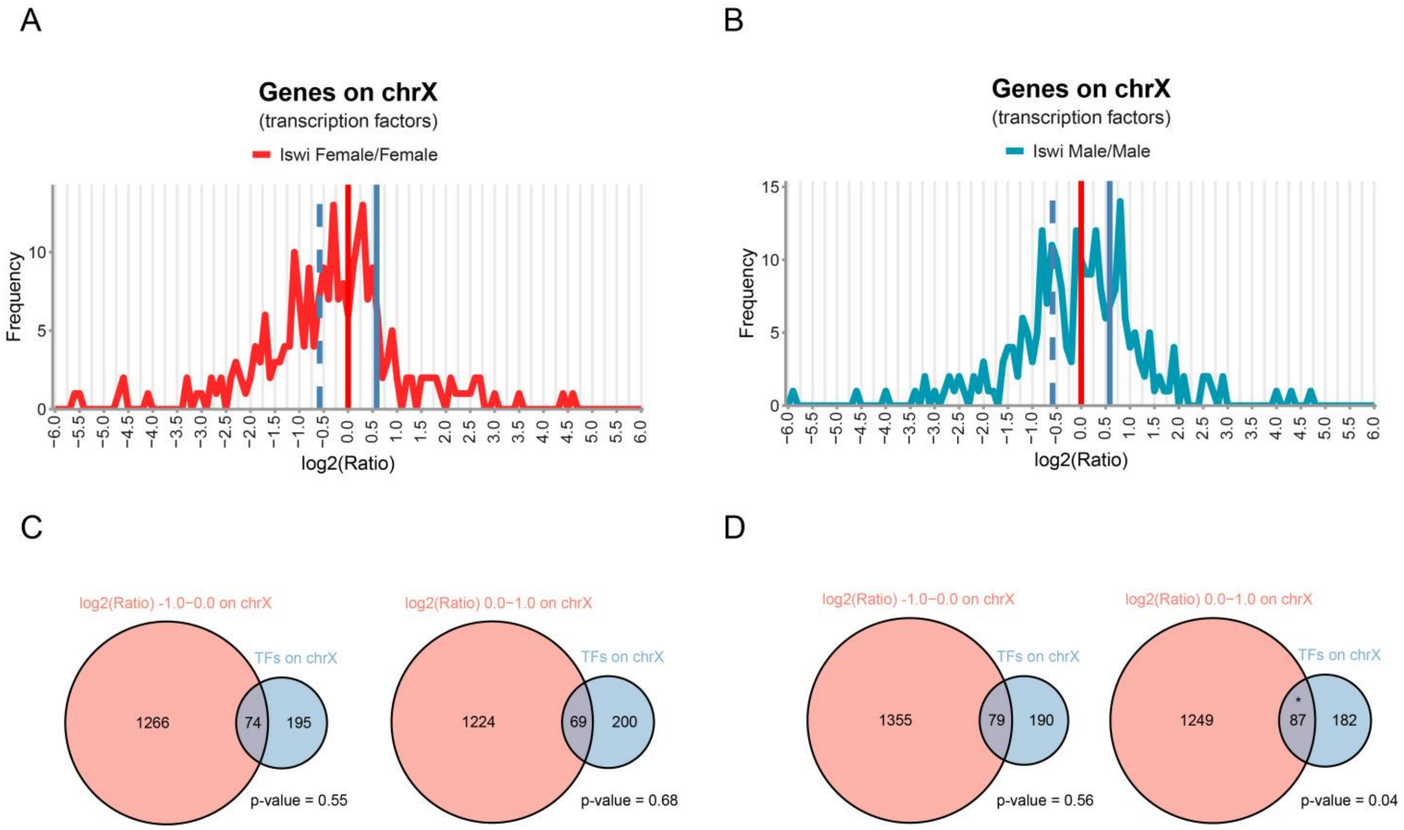

Figure S2 transcription factors expression in ISWI mutant compared to wild-type *Drosophila*. (A-B). Ratio distributions of transcription factors expression of female(A) and male(B) *Drosophila*. (C-D) The Venn diagrams of transcripts with log2(Ratio) values of -1.0-0.0 or 0.0-1.0 and transcription factors on X chromosome of female(C) and male(D) *Drosophila*. Fisher's exact test p values are shown beside the Venn diagrams.

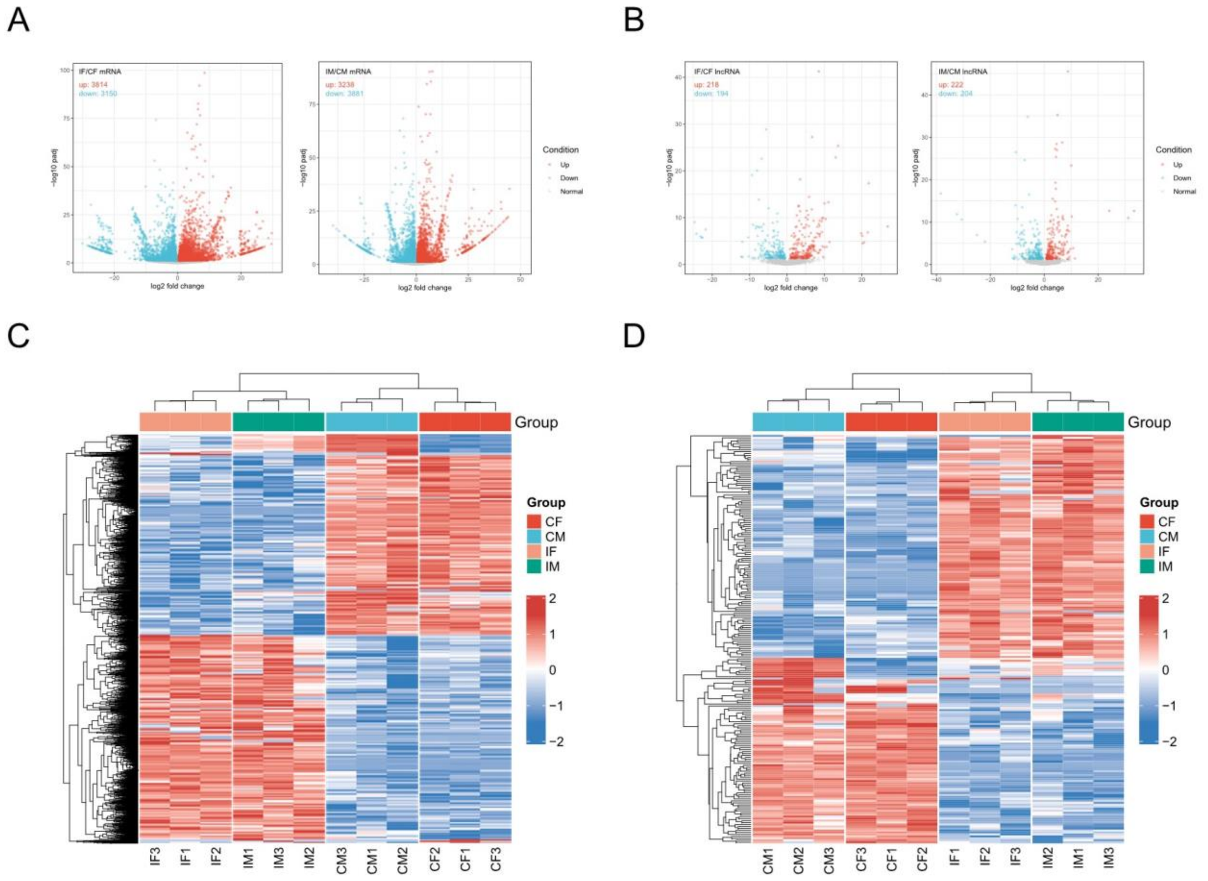

Figure S3 Differentially expressed transcripts of ISWI mutant and wild type. (A.B) Volcano plots of differentially expressed mRNA (A) and lncRNA (B) in ISWI mutation compared to the corresponding sex wild-type drosophila. Differential expressed transcripts are defined as adjusted p-value < 0.05 and fold change >1.25 or <0.8. (C.D) Heatmaps of mRNA (C) and lncRNA (D) of all mutant samples and control groups.

A

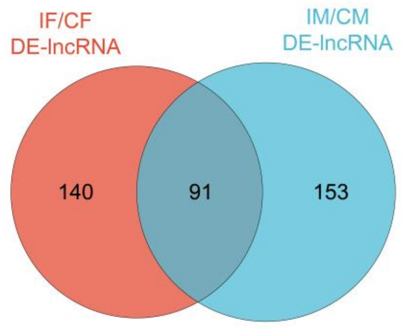

B

lncRNA	GO	GO name
CR31781	GO:0046331	lateral inhibition
CR43306	GO:0007283	spermatogenesis
Hsromega	GO:0000785	chromatin
	GO:0008104	protein localization
	GO:0030162	regulation of proteolysis
	GO:0032270	positive regulation of cellular protein metabolic process
	GO:0035062	omega speckle
	GO:0035063	nuclear speck organization
	GO:0043065	positive regulation of apoptotic process
	GO:0046330	positive regulation of JNK cascade

C

lncRNA transcript	IF_LFC	IM_LFC	lncRNA transcript	IF_LFC	IM_LFC	lncRNA transcript	IF_LFC	IM_LFC	lncRNA transcript	IF_LFC	IM_LFC
lncRNA:CR31781-RD	0.78336722	1.237291252	lncRNA:CR44640-RA	2.745439683	3.079171857	lncRNA:CR46123-RA	8.496015926	8.751432813	lncRNA:CR45551-RA	-2.562840981	-3.350751999
lncRNA:CR43650-RB	0.835045154	2.361658548	lncRNA:CR45150-RA	2.826530438	3.270618314	lncRNA:CR46328-RA	8.707832731	3.398395565	lncRNA:CR43881-RB	-2.393872917	-2.258322891
lncRNA:CR45200-RA	1.053784394	1.895785783	lncRNA:CR45721-RA	2.987920549	5.422025638	lncRNA:CR43417-RA	10.31930449	9.275359546	lncRNA:CR45177-RA	-2.299773356	-2.133067463
lncRNA:CR42862-RC	1.223884927	1.766894817	lncRNA:CR43929-RA	3.133425898	1.863568621	lncRNA:CR44320-RA	-23.12786575	-24.97109337	lncRNA:CR44898-RA	-2.287571958	-1.65919172
lncRNA:CR42862-RB	1.226056564	1.511777172	lncRNA:CR45320-RA	3.164969926	2.356166165	lncRNA:CR44462-RA	-8.321053056	-8.2997713	lncRNA:CR33938-RA	-1.88859909	-2.497180033
lncRNA:CR42874-RA	1.270743246	1.259587128	lncRNA:CR45973-RA	3.170171724	4.714821523	lncRNA:CR43488-RC	-7.890999888	-4.40172107	lncRNA:CR44326-RA	-1.873925777	-2.376063476
lncRNA:CR43459-RC	1.275483053	1.653604919	lncRNA:CR45973-RB	3.494451177	4.300935068	lncRNA:CR45517-RA	-7.531799383	-8.155951753	lncRNA:CR43626-RA	-1.413521568	-1.572881442
lncRNA:CR43651-RA	1.506660026	1.34294082	lncRNA:CR44555-RA	4.118224842	6.620584002	lncRNA:dntRL-RA	-6.575004188	-5.160379302	lncRNA:CR45916-RA	-1.194074346	-1.217972585
lncRNA:CR44526-RA	1.508633381	2.707435519	lncRNA:CR43130-RC	4.211033223	2.600814384	lncRNA:CR43488-RA	-6.372306418	-5.99399048	lncRNA:CR43650-RA	-1.117135692	-0.930991187
lncRNA:CR42719-RB	1.619285885	1.430510857	lncRNA:CR45251-RA	4.680687421	3.362332877	lncRNA:CR40469-RA	-5.588222649	-6.119982253	lncRNA:CR42861-RB	-0.729843412	-0.579643242
lncRNA:CR44938-RA	1.785724338	1.969572652	lncRNA:CR44720-RA	5.176375636	4.139938537	lncRNA:CR44988-RA	-5.288634474	-6.925374524	lncRNA:CR31044-RA	-0.686530123	-0.907799121
lncRNA:CR32582-RH	1.942075495	1.082949934	lncRNA:CR45605-RB	5.307749284	3.290231143	lncRNA:CR45732-RA	-5.169032966	-3.57063053	lncRNA:CR43939-RA	1.377712342	-1.861779431
lncRNA:CR44138-RA	1.970309318	2.046051296	lncRNA:CR9284-RD	5.494250434	7.094395186	lncRNA:Hsromega-RB	-4.919161533	-3.676032738	lncRNA:CR43436-RA	1.564451978	-1.600697248
lncRNA:CR45588-RA	2.103557967	2.035623748	lncRNA:CR45678-RA	5.612933944	5.645555421	lncRNA:CR43967-RA	-4.85132223	-2.268520567	lncRNA:CR44732-RA	2.14001971	-3.031659788
lncRNA:CR45635-RA	2.245322261	3.732004263	lncRNA:CR45181-RA	5.632451161	5.487799316	lncRNA:CR46122-RA	-4.622906009	-2.53442598	lncRNA:CR46216-RD	2.174459842	-1.112797159
lncRNA:CR44311-RA	2.250143851	2.156998482	lncRNA:CR44647-RA	5.671267998	6.266716592	lncRNA:CR45398-RA	-4.162057746	-3.051803685	lncRNA:CR44055-RA	2.380328189	-2.255368424
lncRNA:CR44758-RA	2.255711821	1.788320086	lncRNA:CR45585-RA	5.809964584	4.803561194	lncRNA:CR33218-RC	-4.159721881	-2.124432437	lncRNA:CR44609-RA	4.287875646	-7.549225948
lncRNA:CR44274-RA	2.266253822	3.01373036	lncRNA:CR45348-RA	6.609510248	4.809028174	lncRNA:CR44918-RA	-3.82402273	-1.916219361	lncRNA:CR43279-RA	4.740873027	-2.016909656
lncRNA:CR44197-RA	2.572556435	6.526408124	lncRNA:CR44097-RA	6.715554186	6.676425056	lncRNA:CR43828-RA	-3.519479881	-2.381621504	lncRNA:CR43306-RA	5.155925667	-4.16961761
lncRNA:CR45596-RA	2.573437064	2.971837536	lncRNA:CR45707-RA	6.733195216	3.074784452	lncRNA:CR45312-RA	-3.249310724	-2.727336618	lncRNA:CR44136-RA	6.059847313	-2.217815699
lncRNA:CR44463-RA	2.626801199	3.101536499	lncRNA:CR44645-RA	6.840839344	7.717481238	lncRNA:CR42765-RC	-2.917600241	-3.01311122	lncRNA:CR43279-RB	6.595414018	-3.048182358
lncRNA:CR44458-RA	2.630819397	2.123820997	lncRNA:CR44948-RB	7.028284317	7.118746553	lncRNA:CR45750-RA	-2.787607514	-3.216702133	lncRNA:CR44779-RC	-5.360203425	3.147231179
lncRNA:CR44809-RA	2.727042531	2.561501411	lncRNA:CR45225-RA	7.070724182	3.859020328	lncRNA:CR43242-RA	-2.577493149	-2.603968585			

Figure S4 Differentially expressed known lncRNAs. (A) Differentially expressed known lncRNAs in both sexes; (B) Functions of differentially expressed lncRNAs in both sexes; (C) 91 differentially expressed lncRNAs in both sexes (gray: up-regulated lncRNAs in both sexes; orange: down-regulated lncRNAs in both sexes; yellow: up-regulated in female flies and down-regulated in male flies; blue: down-regulated in female and up-regulated in male flies).

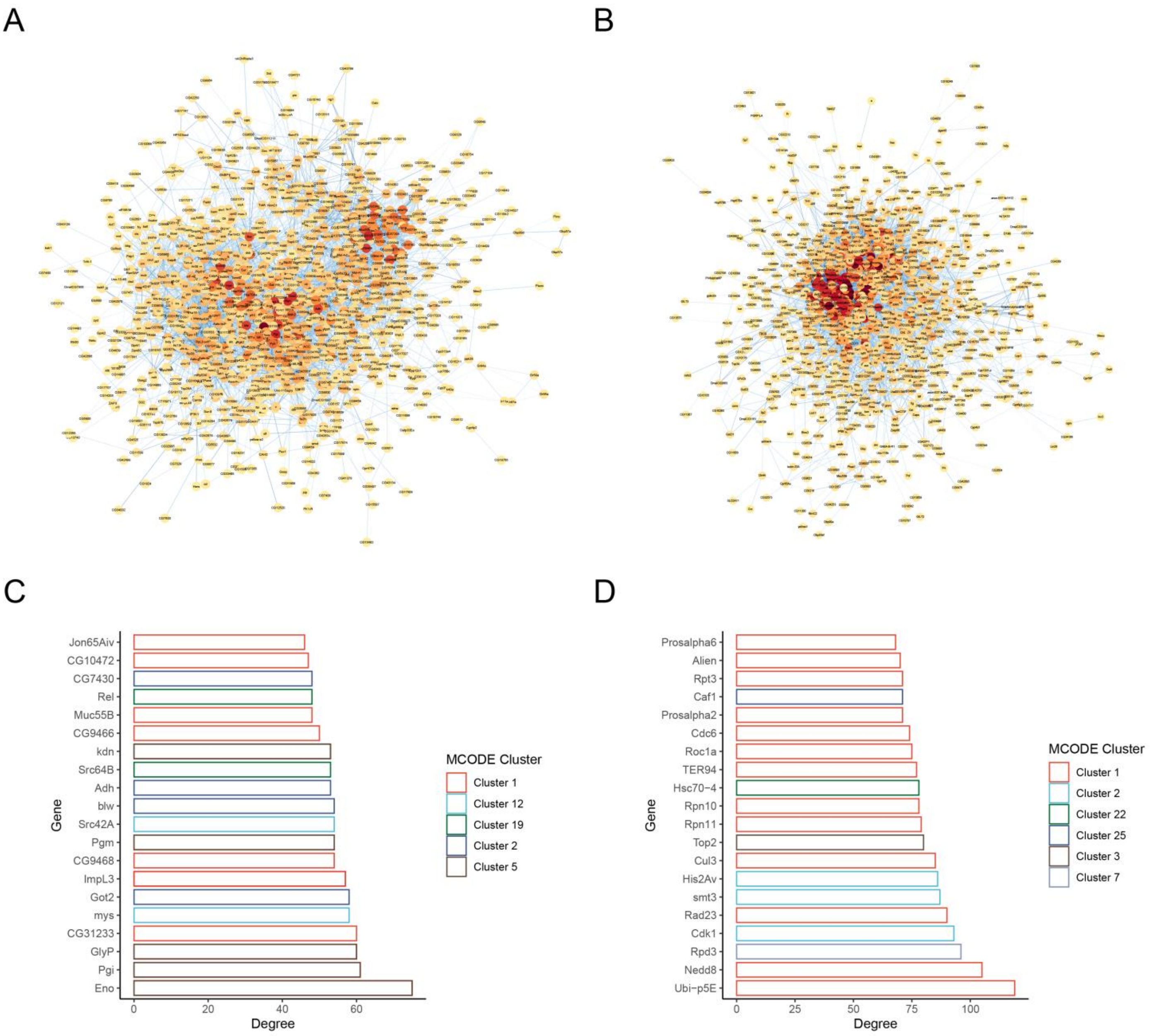

Figure S5 Protein-protein interaction (PPI) network of all DEGs. (A, B) PPI network of all up-(A) or down-(B) regulated DEGs. (C.D) The MCODE-modules of protein-protein interaction (PPI) networks of up- or down-regulates differential expressed genes. The top 20 connectedness genes and their Mcode modules are listed.

A

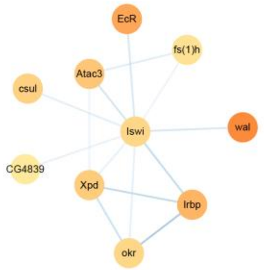

B

Gene	Function	p.adjust
Xpd/fs(1)h/EcR/Iswi/Atac3	positive regulation of nucleic acid metabolic process	4.21E-03
okr/Iswi/Atac3	chromatin remodeling	4.21E-03
Xpd/okr/Irbp	DNA repair	7.97E-03
fs(1)h/csul/EcR	anterior/posterior pattern specification	7.97E-03
EcR/wal	digestive system development	2.96E-02

C

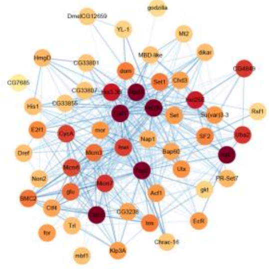

D

Gene	Function	p.adjust
Trl/Mcm7/Su(var)3-3/MBD-like/Nap1/Bap60/glu/SMC2/Mcm6/Top2/Iswi/Set/PR-Set7/His3.3B	DNA conformation change	1.41E-11
Klp3A/Mcm3/Trl/CycA/smt3/glu/mor/SMC2/Mcm6/E2f1/Cdc6/Top2/Ctf4	mitotic cell cycle	1.39E-06
Utx/His2Av/EcR/Trl/Su(var)3-3/Bap60/mor/E2f1/mbf1/Iswi/Dref	positive regulation of RNA biosynthetic process	1.81E-05
Hel25E/Su(var)3-3/dom/Nap1/Chrac-16/Bap60/Chd3/mor/Iswi/YL-1	chromatin remodeling	4.02E-09
Mcm3/gkt/EcR/smt3/glu/E2f1/mbf1	central nervous system development	1.04E-03

E

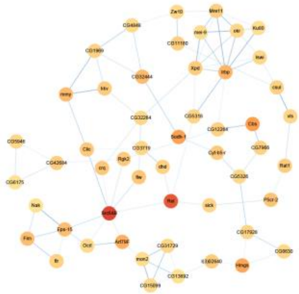

F

Gene	Function	p.adjust
flw/CG32264/mon2/Fim/Ocrl/sick/Src64B/flr	actin cytoskeleton organization	4.11E-03
Ku80/mei-9/CG5316/Irbp/okr/Xpd/Rel	cellular response to DNA damage stimulus	4.11E-03
crq/Arf79F/Nak/Ocrl/Eps-15/CG31729	endocytosis	4.22E-03
mei-9/dhd/Zw10/okr/Arf79F/Src64B	meiotic cell cycle process	1.34E-02
flw/csul/vls/mon2/Arf79F	developmental maturation	3.09E-02

G

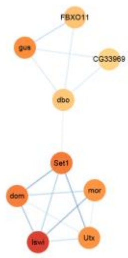

H

Gene	Function	p.adjust
FBXO11/Iswi/mor/dom	chromatin remodeling	4.56E-04
Utx/mor/dom	negative regulation of cell population proliferation	1.95E-03
Set1/Utx/dom	histone modification	1.04E-02
CG33969/FBXO11/gus	proteasomal protein catabolic process	1.27E-02
gus/Utx/mor	instar larval or pupal morphogenesis	3.02E-02

Figure S6 Protein interaction networks associated with ISWI. (A-D) Protein interaction networks that directly interact with ISWI from the up-regulated(A) and down-regulated(C) protein interaction diagrams and functional annotations of these genes (B.D). (E-H) Protein interaction networks made by MCODE software package from the up-regulated(E) and down-regulated(G) protein interaction diagrams. The functional annotations of these genes list on the left (F.H).

A

	Gene	Degree	Function
Transcription	Bin1	58.0	Interacts with sequence-specific transcription factors to help recruit the Sin3A-HDAC1 histone deacetylase co-repressor complex to target genes [30].
	Su(var)2-HP2	51.0	Associated with centromeric heterochromatin, telomeres and the fourth chromosome [31].
	E(bx)	45.0	Histone-binding component of NURF [32].
	sisA	30.0	Activates transcription of the master feminizing switch gene Sxl specifically in diplo-X somatic cells [33]. Proper embryonic midgut development in both sexes.
Ovarian development	Ras85d	13.0	Acts downstream of several cell signals, mainly from receptor tyrosine kinases, to regulate tissue growth and development. [34]

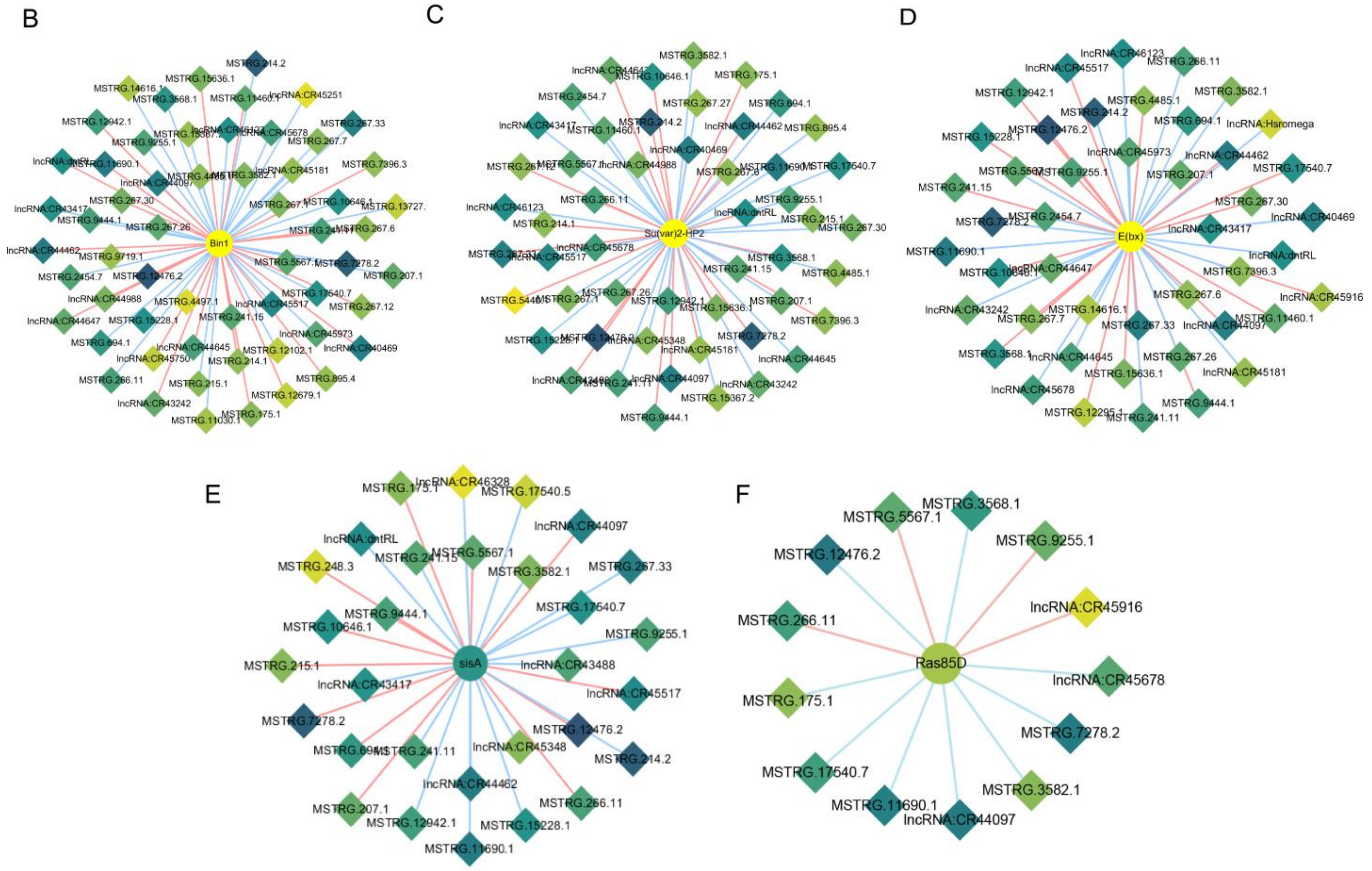

Figure S7 Possible interactors for DE-lncRNAs. (A) Five possible target genes and their connection degree (the number of co-expressed lncRNAs or mRNAs with it) and main functions. (B-F) The five genes in Figure A and their co-expressed lncRNAs: Bin (B), Su(VAR) 2-HP2 (C), E(bx)(D),sisA (E) and Ras85D(F). Network diagram of me31B and co-expressed lncRNAs. The diamond nodes represent lncRNAs, and the circle nodes represent mRNAs. The darker the color of node, the higher the degree of connection (the more co-expressed interactions). The diamond nodes represent lncRNAs, and the circle nodes represent mRNAs. The darker the color of node, the higher the degree of connection (the more co-expressed interactions). The red edge represents a positive correlation and the blue edge represents a negative correlation.

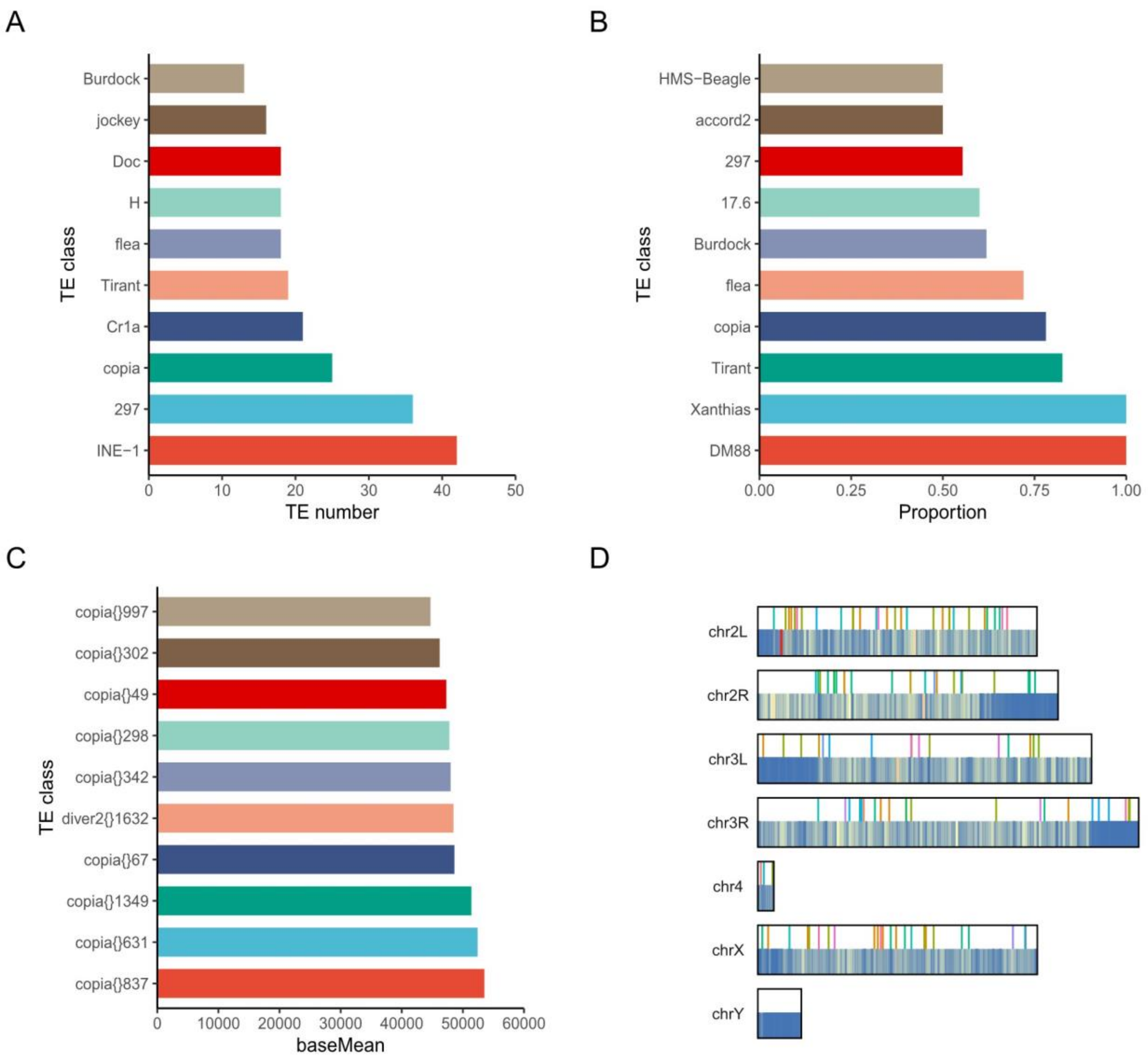

Figure S8 Characteristics and chromosomal localization of transposons with differential expression. (A) Top 10 TE class by number of transposons. (B) Top 10 TE class by the proportion of DE-TEs in the TE class. (C) Top 10 transposons in terms of expression in all samples. The horizontal coordinate baseMean is the amount of expression processed and standardized by DEseq2. (D) The location of TE class on chromosomes. The upper part of the white is the TEs on the chromosome, and the lower part of the blue is the gene on the chromosome.

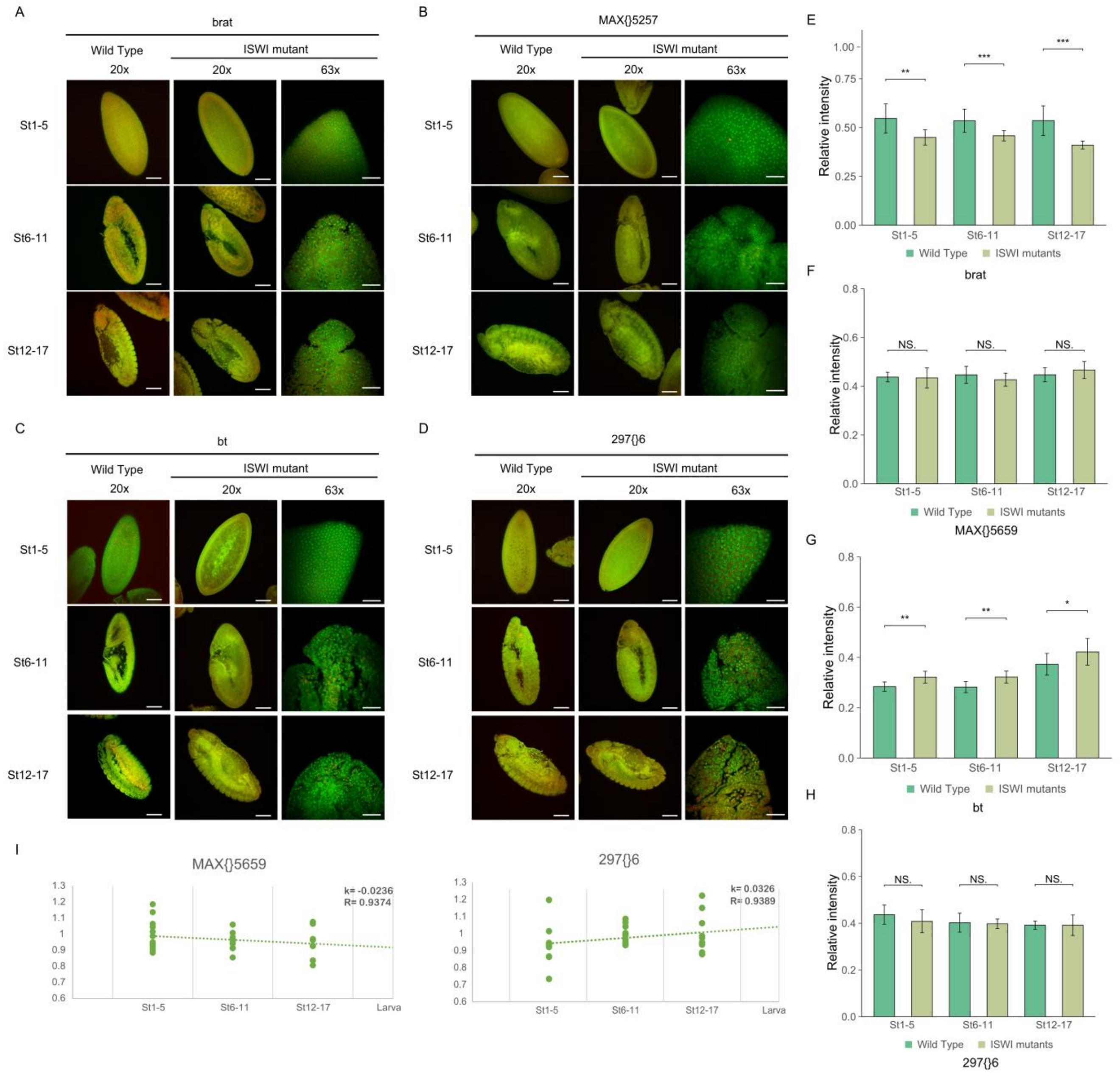

Figure S9 Embryo-FISH of two genes and co-expressed transposons. (A-D) brat(A), MAX{}5659(B), bt(C) and 297()6 RNA expression patterns in total embryo preparations. The genotype of the sample is shown in the horizontal axis above, and the development stage of the sample is shown in the left vertical axis. The red pseudo-color is the signal from Probe and the green pseudo-color is the signal from nucleus. Scale bars, 80 µm. RNA subcellular Location Patterns in mutant *Drosophila*. Scale bars, 30 µm. (E-H) The RNA brat(E), MAX{}5659(F), bt(G), 297{}6(H) relative fluorescence intensity of ISWI mutants and wild-type RNA signals was determined by comparing them with DAPI signals. The asterisk indicates $p < 0.05$ for the two-tailed student t test. (I) The ratio of transposon MAX{}5659 and 297{}6 expression in ISWI mutants to that in wild-type at three stages of embryonic development.
